# Naphthazarin Derivatives in the Light of Intra- and Intermolecular Forces

**DOI:** 10.3390/molecules26185642

**Published:** 2021-09-17

**Authors:** Karol Kułacz, Michał Pocheć, Aneta Jezierska, Jarosław J. Panek

**Affiliations:** Faculty of Chemistry, University of Wrocław, ul. F. Joliot-Curie 14, 50-383 Wrocław, Poland; karol.kulacz@chem.uni.wroc.pl (K.K.); michal.pochec@chem.uni.wroc.pl (M.P.)

**Keywords:** intramolecular hydrogen bonds, gas phase, crystalline phase, DFT, MP2, CCSD, AIM, SAPT, nuclear quantum effects, CPMD

## Abstract

Our long-term investigations have been devoted the characterization of intramolecular hydrogen bonds in cyclic compounds. Our previous work covers naphthazarin, the parent compound of two systems discussed in the current work: 2,3-dimethylnaphthazarin (**1**) and 2,3-dimethoxy-6-methylnaphthazarin (**2**). Intramolecular hydrogen bonds and substituent effects in these compounds were analyzed on the basis of Density Functional Theory (DFT), Møller–Plesset second-order perturbation theory (MP2), Coupled Clusters with Singles and Doubles (CCSD) and Car-Parrinello Molecular Dynamics (CPMD). The simulations were carried out in the gas and crystalline phases. The nuclear quantum effects were incorporated a posteriori using the snapshots taken from ab initio trajectories. Further, they were used to solve a vibrational Schrödinger equation. The proton reaction path was studied using B3LYP, ωB97XD and PBE functionals with a 6-311++G(2d,2p) basis set. Two energy minima (deep and shallow) were found, indicating that the proton transfer phenomena could occur in the electronic ground state. Next, the electronic structure and topology were examined in the molecular and proton transferred (PT) forms. The Atoms In Molecules (AIM) theory was employed for this purpose. It was found that the hydrogen bond is stronger in the proton transferred (PT) forms. In order to estimate the dimers’ stabilization and forces responsible for it, the Symmetry-Adapted Perturbation Theory (SAPT) was applied. The energy decomposition revealed that dispersion is the primary factor stabilizing the dimeric forms and crystal structure of both compounds. The CPMD results showed that the proton transfer phenomena occurred in both studied compounds, as well as in both phases. In the case of compound **2**, the proton transfer events are more frequent in the solid state, indicating an influence of the environmental effects on the bridged proton dynamics. Finally, the vibrational signatures were computed for both compounds using the CPMD trajectories. The Fourier transformation of the autocorrelation function of atomic velocity was applied to obtain the power spectra. The IR spectra show very broad absorption regions between 700 cm${}^{-1}$–1700 cm${}^{-1}$ and 2300 cm${}^{-1}$–3400 cm${}^{-1}$ in the gas phase and 600 cm${}^{-1}$–1800 cm${}^{-1}$ and 2200 cm${}^{-1}$–3400 cm${}^{-1}$ in the solid state for compound **1**. The absorption regions for compound **2** were found as follows: 700 cm${}^{-1}$–1700 cm${}^{-1}$ and 2300 cm${}^{-1}$–3300 cm${}^{-1}$ for the gas phase and one broad absorption region in the solid state between 700 cm${}^{-1}$ and 3100 cm${}^{-1}$. The obtained spectroscopic features confirmed a strong mobility of the bridged protons. The inclusion of nuclear quantum effects showed a stronger delocalization of the bridged protons.

## 1. Introduction

The nature of hydrogen bonds is complex and still presents open questions. In addition to conventional hydrogen bonds, during recent decades so called unconventional hydrogen bonds have appeared as important scientific topics [1,2,3,4,5,6,7,8]. Hydrogen bonds are located on the interaction strength ladder in the middle position, between covalent, ionic, and van der Waals interactions [9]. They are much weaker than chemical covalent bonds, but their presence is of great significance in nature. The strength of most hydrogen bonds is between 10 kJ/mol and 40 kJ/mol [10]; however, they are ubiquitous and cannot be neglected in the discussion of factors decisive for the structure of bulk materials: liquids and solid states. They are key elements of many processes at the molecular level, as well as influencing molecular and macroscopic properties of various systems [11,12,13,14,15]. They were found to be, e.g., present in enzymatic reactions [16,17], responsible for structure stabilization [18,19,20,21,22], arrangement of molecules in crystals [23,24] and supporting molecular engineering [25,26]. Therefore, it is evident that hydrogen bonds are non-covalent interactions relevant in various branches of contemporary science [27].

Hydrogen bonds are generally divided into intra- and intermolecular ones. While the latter provide the possibility of supramolecular assembly and molecular recognition, the former are relevant to the molecular structure, stabilizing the hydrogen-bonded conformations and giving rise to the tautomeric forms. The intramolecular hydrogen bonds are more open to types of enhancement such as resonance-assisted phenomena [28,29], but on the other hand the charge-assisted bonds can be easily formed between two separate molecules [30,31,32]. Another aspect related to hydrogen bonds is the proton transfer phenomenon and associated changes in geometric and electronic structure parameters [33,34,35]. This phenomenon has been studied using experimental and theoretical approaches, because its role cannot be neglected in biomolecules and other compounds where tautomeric forms occur, e.g., see refs. [36,37,38,39,40,41,42].

The current study is a continuation of our long-term research effort to shed light on the hydrogen bridge dynamics in molecular crystals with diverse chemical compositions—cyclic compounds in particular. In order to make our study comprehensive, we focus not only on intramolecular interactions, but also on intermolecular forces. Our studies covered, e.g., description of molecular properties in monocyclic aromatic o-hydroxy Schiff and Mannich bases [43,44], bicyclic N-oxides [45,46,47] and proton sponges [41,48]. Following the line of our research devoted to intramolecular hydrogen bonds investigations in compounds possessing fused rings, we have focused our attention on naphthazarin and its derivatives. The 5,6-dihydroxy-1,4-naphtoquinone (commonly referred to as naphthazarin) and its derivatives are a part of the wider naphthoquinone group. Members of this class of compounds are widely distributed in natural sources [49] and have been proven to possess many interesting biological properties. Recent years have brought reports on their antibacterial, antifungal and biostatic activities [50,51]. Naphthazarin, alone or in conjunction with other compounds, can be used as a potent biopesticide or insecticide [52]. Medicine also has many potential ways of utilizing naphthazarin and its derivatives. They have been proven to possess high anti-inflammatory potential and the positive effect on the healing of wounds [53]. More elaborate derivatives can also be applied in oncological treatment—they have been reported to have an inhibitory effect on DNA Topoisomerase-I [54], heat-shock factor and glutathione status in the aftermath of hypoxia [55]. All of the aforementioned properties show that there is still need to study both the possible ways of synthesis and design of derivatives with desired properties [56,57]. One of the most pronounced characteristics of naphthazarin is the presence of two hydrogen bonds between the hydroxyl groups and their neighboring carbonyl oxygen atoms. This not only forms two distinct quasi-rings in the structure, but also allows the opportunity to study the effects of substitution in the fused rings and double hydrogen bonding properties [58]. In case of the naphthazarin and its selected derivatives, investigations into physico-chemical properties are reported by [59,60,61]. The computational studies have been also used to assess compatibility of experimental and theoretical data in the IR and Raman spectra of naphthazarin, which in turn allowed precise assignment of bands [62].

**Figure 1:**
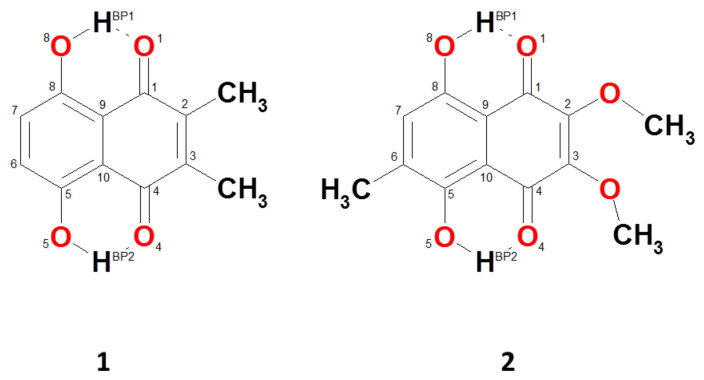
Molecular structures of the studied 2,3-dimethylnaphthazarin (**1**) and 2,3-dimethoxy-6-methylnaphthazarin (**2**) with the atom numbering scheme applied in the current study.

Here, we present our theoretical results obtained for two naphthazarin derivatives: 2,3-Dimethyl-5,8-dihydroxy-1,4-naphthoquinone (**1**) and 5,8-Dihydroxy-2,3-dimethoxy-6-methylnaphtho-1,4-quinone (**2**), presented in Figure 1 and Figure S1. The motivation for the current work and the choice of the compounds was the comparison of symmetric and asymmetric substitution with diverse, but not very strong, substituent properties represented by the classical physico-chemical parameters, Hammett constants. The –Me and –OMe substituents possess different properties regarding their electro- and nucleophilicity. The methyl groups are relatively mild on the Hammett scale (their classical Hammett constants are only −0.07 and −0.17 for $\sigma_{m}$ and $\sigma_{p}$, respectively [63]. The –OMe substituents are reported to reach the values of +0.12 and −0.27 for $\sigma_{m}$ and $\sigma_{p}$ [63]. Such values indicate the possibility of an interesting interplay between local properties of the substituents and an environmental influence in the solid state (with the presence of other molecules). The physico-chemical properties of both compounds were studied on the basis of X-ray methods as well as, e.g., NMR spectroscopy for **1** by Rodríguez et al. [64] and for **2** by Cannon et al. [65]. Concerning compound **1** the crystal structure is built of molecules, which are stacked up the c axis and the molecules overlap forming the charge-transfer complex [64]. Compound **2** contains two methoxyl groups, which are slightly different with regard to geometry: one of the groups lies in the plane of the ring, but the methyl group of the methoxyl deviates from the ring plane by 1.08 Å [65]. This experimentally observed difference in geometry, which may result from packing forces, would not necessarily be detected in solution. According to the authors, electronic effects were responsible for observed non-equivalence of the methoxyl resonances in the NMR spectrum in solution. The electron-donating properties of the methyl group are well known and characterized [63]. Inductive effects usually cover short distances; however, in the case of compound **2**, they could influence the methoxy groups as well [65]. Therefore, the main aim of the study was further examination of internal and external forces responsible for the molecular features of the compounds.

In order to achieve the goals delineated in the previous paragraphs, the fundamental issues of comparisons of intra- and intermolecular phenomena, influence of substituents, and correlation of bridge proton motions, diverse theoretical approaches were considered and employed. Static and dynamical models were developed on the basis of Density Functional Theory (DFT) [66,67] and Car–Parrinello molecular dynamics [68]. The simulations were carried out in the gas phase and in the solid state. Our particular attention was focused on: (i) The proton reaction path and related energy changes in the monomeric forms of the studied compounds. We put emphasis on the substituent effects on the hydrogen bond properties; (ii) The electronic structure and topology changes—the comparison of molecular and proton transferred (PT) forms on the basis of the Atoms in Molecules (AIM) theory [69]; (iii) The energy partitioning in the dimers extracted from the X-ray data as well as obtained theoretically on the basis of Symmetry-Adapted Perturbation Theory (SAPT) [70]; (iv) The hydrogen bridges dynamics in the gas and crystalline phases, which enabled us to detect differences derived from environmental effects—gas phase vs. solid state comparison and the structural impact of the nuclear quantum effects (NQE) for the bridge protons; (v) Vibrational signatures present in the studied compounds, but particularly associated with the intramolecular hydrogen bond—gas phase vs. solid state comparison. To the best of our knowledge this is the first study that examines these particular naphthazarin derivatives considering intra- and intermolecular forces.

## 2. Results and Discussion

### 2.1. Geometric and Electronic Structure Description of Naphthazarin Derivatives Monomers with Special Emphasis on Intramolecular Hydrogen Bonds

This section contains results from diverse theoretical approaches, ranging from gradient-corrected DFT to post-Hartree–Fock schemes. However, the common trait is that the computational models represent the gas phase molecules using atom-centered Gaussian basis sets providing wavefunctions, Kohn–Sham orbitals and electron density. The chosen DFT functionals belong to the most widely used approaches: the PBE functional is of the Generalized Gradient Approximation (GGA) type and does not use the exact (Hartree–Fock) exchange. B3LYP is a hybrid functional with Hartree–Fock admixture. The ωB97XD is also a hybrid functional, additionally including empirical dispersion correction. The chosen post-Hartree–Fock schemes are Møller–Plesset second order perturbative calculus, MP2, and Coupled Cluster theory with single and double excitations, CCSD. The Car–Parrinello scheme, relevant to the further sections, is based on the delocalized plane-wave basis sets with inherent periodicity. This makes CPMD calculations technically easier for solids and liquids than for the gas phase, where periodicity has to be artificially removed. Moreover, exact (Hartree–Fock) exchange is not easily implementable on the plane-wave basis. Therefore, CPMD calculations utilize almost solely the GGA functionals, such as PBE, due to their efficiency and well tested performance.

The molecular structures of the monomeric forms of studied 2,3-dimethylnaphthazarin (**1**) and 2,3-dimethoxy-6-methylnaphthazarin (**2**) are presented in Figure 1. The intramolecular non-covalent interactions present in the molecules are classified as Resonance-Assisted Hydrogen Bonds (RAHBs) [28]. The geometry of the studied compounds was modified to analyze the energy of various isomers (see Table S1 for details; this table includes not only the electronic energy, but also the energy values corrected for the vibrational zero point energy (ZPE) contribution). The lowest energy was found for the molecular forms. The double-PT forms are slightly higher in energy (we will use the convention: electronic/ZPE-corrected value in kcal/mol): for **1**, the difference is 1.94/1.91 kcal/mol (B3LYP), 1.61/1.38 kcal/mol (PBE) and 2.02/1.54 kcal/mol (ωB97XD). For **2**, the corresponding increases in energy of the double-PT form with respect to the molecular form are: 2.94/2.74 kcal/mol (B3LYP), 2.78/2.29 kcal/mol (PBE) and 2.91/2.58 kcal/mol (ωB97XD). This shows that from a thermodynamic point of view the molecular forms are preferred, but not strongly, over the PT forms. The trans forms through which the double-proton transfer proceeds [61] are even higher in energy, e.g., for **1** the differences with respect to the molecular forms are: 5.30/4.65 kcal/mol (B3LYP), 2.82/1.26 kcal/mol (PBE) and 6.81/6.23 kcal/mol (ωB97XD). However, these values are all well within the range thermally accessible to the extended molecules at room temperature, especially when the effects of donor–acceptor distance modulation are accounted for (see the Car–Parrinello study below).

The geometric details of the intramolecular hydrogen bonds (comparison of experimental and computational data) are summarized in Table S2. It is shown that the DFT method was able to reproduce the metric parameters related to the hydrogen bonding with a good agreement. The impact of the substituents, as already noted in the Introduction, corresponds to their inductive and resonance properties. The Hammett constants are, respectively: $\sigma_{m}$= −0.07 and $\sigma_{p}$= −0.17 for the -Me, while for –OMe they are $\sigma_{m}$= +0.12 and $\sigma_{p}$= −0.27 [63]. The bridge protons are located at the non-substituted ring of **1** and methyl-substituted ring of **2**, which shows that substitution affects aromaticity and the modified rings prefer participation in the quinoid-like structure.

**Figure 2:**
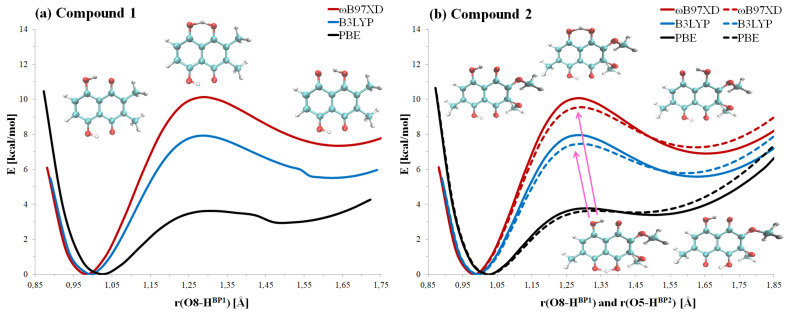
The potential energy profiles for the proton motion in the hydrogen bridges of compounds **1** (**a**) and **2** (**b**), respectively. The hydrogen bridges denoted as O8-H${}^{BP1}$...O1 (Bridge 1) for compound **1** and O8-H${}^{BP1}$...O1 (Bridge 1) and O5-H${}^{BP2}$...O4 (Bridge 2) for compound **2** are presented. In the case of **2**, the solid line denotes Bridge 1 while the dotted line denotes Bridge 2.

The proton potential functions for the proton motion are presented in Figure 2. The proton reaction energy paths were investigated using B3LYP, ωB97XD and PBE functionals with the 6-311++G(2d,2p) basis set. As shown in the figure, two energy minima were obtained in the case of both compounds and both studied bridges. In the case of compound **1**, only one hydrogen bridge was analyzed due to the symmetry exhibited by the compound. Concerning compound **2**, both intramolecular hydrogen bridges were analyzed. The compound is not symmetrical due to the presence of the methyl group. Before discussing the details of the proton potential functions, it is necessary to consider whether single- or double-proton transfer should be pursued. Our DFT and CPMD results for naphthazarin [61] indicate that the simultaneous double-proton transfer is less probable and leads to higher barriers, which is assumed to be the effect of deeper modification of aromaticity than in the case of the single-proton transfer event. However, the single-proton transfer enables the second proton transfer (PT) event to happen very fast but not simultaneously, in the order of several O-H stretching periods.

The deeper energy minimum is localized at ca. 1 Å of the O8-H${}^{BP1}$/O5-H${}^{BP2}$ covalent bond length in both compounds. The elongation of the bond towards the acceptor atom (O1/O4) provided information of the energy barrier, which was found—depending on the applied functional—to be very similar in both studied compounds. The highest energy barrier was obtained for the ωB97XD functional (10.1 ± 0.05) kcal/mol for Bridge 1 of both compounds and (9.55 ± 0.05) kcal/mol for Bridge 2 of compound **2**. The results obtained with assistance of the B3LYP functional are (7.95 ± 0.05) kcal/mol for Bridge 1 and (7.45 ± 0.05) kcal/mol for Bridge 2 in the case of compound **2**. The lowest energy barrier was noticed as a result of PBE functional application (3.55 ± 0.05) kcal/mol for all cases. The DFT results were validated with the single-point energy calculations at the post-Hartree–Fock MP2 and CCSD level for the PBE geometries. Both of these approaches yielded the same ordering of relative energies than the DFT functionals: the barriers for Bridge 1 are almost equal for **1** and **2**, and the barrier for Bridge 2 of compound **2** is slightly lower. The MP2 perturbative calculus provided a barrier height of 6.72 kcal/mol for **1**, 6.86 kcal/mol for Bridge 1 of **2**, and 6.59 kcal/mol for Bridge 2 of **2**. The corresponding CCSD values of barrier height estimate are: 9.59 kcal/mol for **1**, 9.37 kcal/mol for Bridge 1 of **2**, and 9.13 kcal/mol for Bridge 2 of **2**. Our previous results on naphthazarin [61] have shown that MP2 and CCSD methods provide PT barrier heights correspondingly lower and higher from the accurate CCSD(T) barrier height, and we expect similar performances from these methods in the current study. This indicates that the barrier height estimates from the post-Hartree–Fock methods and DFT functionals are in agreement.

Returning to the discussion of structure–energy relations, we note that the second energy minimum is shallow; therefore, we could expect that the bridged proton is mostly localized on the donor (O8/O5) atom. However, the presence of the second energy minimum indicates that the bridge protons are labile and they could approach the proton-acceptor atom domain. Almost identical energy barriers showed that the substituent effects as well as the lack of the symmetry (in the case of compound **2**) have not significantly influenced the proton transfer reaction path in the investigated napththazarin derivatives.

**Figure 3:**
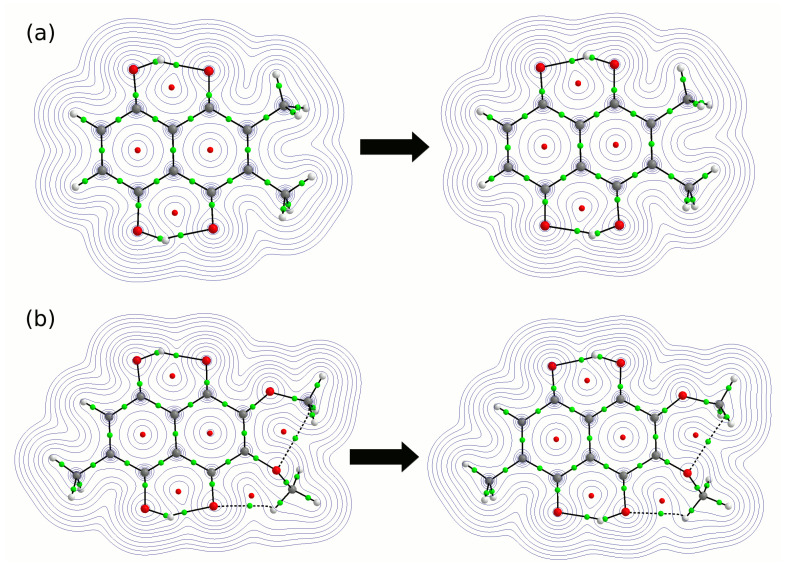
Topology maps of electron density obtained on the basis of AIM theory at the B3LYP/6-311++G(2d,2p) level of theory for compounds **1** (**a**) and **2** (**b**). The molecular (left) and proton transferred (right) forms are presented. The black solid and dashed lines indicate the intramolecular interaction paths. The green and red dots mark the presence of BCPs and RCPs, respectively.

The electronic structure analysis was carried out based on AIM theory. The selected results of the analysis are presented in Table 1 and Table 2. The partial atomic charges are reported for atoms forming quasi-rings in the studied compounds (see Table 1). We have analyzed molecular and tautomeric (proto transferred (PT)) forms of both compounds. The electron density of the donor atom (O8/O5) is smaller when the bridged proton is attached to it. A decrease in the electron density at the acceptor atom (O1/O4) is observed for the tautomeric (PT) form. It can be seen that the hydrogen atoms (H${}^{BP1}$ and H${}^{BP2}$) are more positively charged when they are transferred to the acceptor atom side. Next, the sum of partial atomic charges in the quasi-rings was computed. It was found that for compound **1**, Bridge 1, the sum decreased from −0.1532 [e] in the molecular form to −0.1591 [e] in the proton transferred form. A similar observation was made for compound **2**—the sum of the quasi-ring (Bridge 1) atomic charges decreased from −0.1042 [e] to −0.1089 [e]. Concerning the hydrogen bridge denoted as Bridge 2 (see Table 1), in compound **1**, there was a decrease in the sum of the partial atomic charges in the quasi-ring from −0.1554 [e] to −0.1583. However, an opposite situation was found in the case of compound **2**—there was an increase in the values of the sum of the atomic charges from −0.1707 to −0.1663 [e]. This could be associated with the presence of the methyl group in the vicinity of the quasi-ring and asymmetry introduced by it to compound **2**. It is also known from the crystal structure of the compound [65] that the methoxy groups are sterically not equivalent; moreover, these groups are not chemically equivalent due to their having different relative positions with regard to the methyl group. There was also an interaction between the methoxy group and O4 proton-acceptor atom (for details, see the text below). The interatomic O8...O1 and O5...O4 distances determined experimentally are equal to 2.551 Å and 2.589 Å, respectively. This could also be the reason why an opposite tendency concerning the electron density distribution was observed for compound **2** (Bridge 2). The values of electron density and its Laplacian at Bond Critical Points (BCPs) of intramolecular hydrogen bonds for both compounds are shown in Table 2. The electron density values at the hydrogen bridge BCPs are consistent with our previous calculations performed for 2,3-dichloronaphthazarin [71]. The covalent O8-H${}^{BP1}$/O5-H${}^{BP2}$ bonds are stronger than those formed after proton transfer (O1-H${}^{BP1}$/O4-H${}^{BP2}$). This observation was made after the electron density and its Laplacian examination at BCPs. The electron density values at BCPs are higher for the OH covalent bonds in the molecular forms of compounds **1** and **2**. However, the intramolecular hydrogen bonds are stronger (higher electron density values at BCPs) for the proton transfer (PT) forms. The values at BCPs obtained based on AIM theory are rather similar for molecular and PT forms. They do not much differ, even comparing compound **1** with compound **2**. This could suggest that the proton transferred form is best described not as O${}^{-}$...${}^{+}$H-O, but as simply O...H-O, in parallel with the molecular form O-H...O. The topology maps of electron density are presented in Figure 3. They contain molecular properties common for the AIM description of the electronic structure: critical points (BCPs and RCPs), which are stationary points of the electron density field (i.e., the density gradient is zero at the critical point). In the graphical presentation of Figure 3, these critical points are recognizable as maxima (nuclear positions), saddle points, and minima. Due to the presence of intramolecular hydrogen bonds, the typical quasi-rings were formed and recognized by the BCPs of covalent bonds and the indicated bond paths of the hydrogen bridges. In addition, in the case of compound **2**, some intramolecular contacts were detected between the methoxy groups as well as between the hydrogen of the methoxy group with the O4 atom from the second hydrogen bridge. The presence of an intramolecular hydrogen bonds stabilizes the conformation of the molecules. However, the topology maps showed (in the case of compound **2**), that the electron density distribution in the hydrogen bridge (Bridge 2) could be affected by the competitive interactions introduced by the methoxy substituents. As is shown, two additional quasi-rings were found, indicating that the C-H...O intramolecular hydrogen bonds were formed. They are characterized by the presence of the BCPs and RCPs. However, the presence of such intramolecular interactions was not identified experimentally in the crystal structure [65]. Therefore, the presence of the interactions could be driven by steric effects and degrees of freedom introduced to the isolated molecule model.

### 2.2. Intermolecular Forces in Naphthazarin Derivatives Dimers Based on Symmetry-Adapted Perturbation Theory (SAPT)

**Figure 4:**
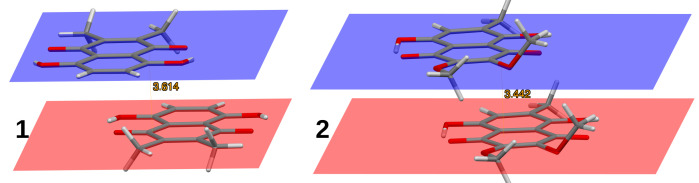
Two distinct types of stacking in the crystal structures of **1**, 2,3-dimethylnaphthazarin (anti-parallel stacking), and **2**, 2,3-dimethoxy-6-methylnaphthazarin (parallel stacking). The interplanar distances are 3.614 Å and 3.442 Å, respectively.

The presence of two distinct types of stacked dimers in the crystals of **1** or **2** (anti-parallel vs. parallel arrangement of molecules, respectively; see Figure 4) indicates that even the relatively mild substitution can influence crystal packing forces. It is therefore necessary to investigate the molecules of **1** and **2** on the basis of interaction energy partitioning schemes. Symmetry-Adapted Perturbation Theory (SAPT, see Ref. [70]) has become a de facto standard for such investigations, although many other approaches exist, some of them capable of tackling covalent bonding, for example, a DFT-based energy decomposition analysis [72] or localized orbital energy decomposition, LMOEDA, useful for non-covalent forces such as beryllium bonds [73]. While analyzing the results presented in this section, it is necessary to remember that SAPT is a perturbative approach, in which intra- and intermonomer correlations are treated separately. SAPT0 omits the intramonomer correlation, while SAPT2 includes this effect up to the second order of perturbation. Both levels account for the intermonomer electron correlation, which is the source of polarization and dispersion contributions. The SAPT partitioning divides the interaction energy into “static” contributions (electrostatic interaction of frozen electron densities, and Pauli exchange repulsion) and “correlated” terms (induction–mutual polarization of monomers—and dispersion).

**Figure 5:**
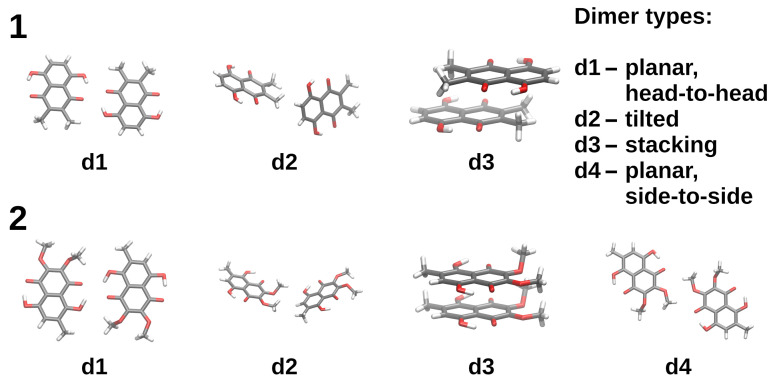
Dimers extracted from the crystal structures of compounds **1** (upper part) and **2** (lower part), used in the SAPT study.

The crystal structures of both compounds contain the following basic types of dimeric structures, depicted in Figure 5: d1—the head-to-head planar arrangements, d2—molecular planes tilted at a shallow angle, d3—stacking, d4—planar arrangement with a side-to-side skew (present only for **2**). Direct use of the experimental structures in the SAPT calculations leads to the results gathered in Table 3, while the DFT-optimized structures are described in Table 4. It must be stressed that the DFT optimization leads to the collapse of the d2-type dimers into the stacking arrangement, underlining the role of the confinement of molecules leading to the formation of diverse structural motifs.

The results gathered in Table 3 and Table 4 show that the energetically most important structural motif (stacking dimer d3) is formed with the dominant role of dispersion. The role of dispersion is visible especially when the d3 dimers of experimental solid state structure are compared with their DFT-optimized analogues. Surprisingly, the latter are more strongly bound. This is an outcome of two competing factors. On the one hand, the crystal electrostatic and steric field tends to squeeze the molecules together, so that no empty voids remain in the structure. This promotes smaller intermolecular separations and stronger stacking forces. On the other hand, the presence of neighbouring molecules means that the capacity of the molecule to interact with its neighbours must split between much more interactions than in the dimer. The latter factor prevails, and the DFT-optimized stacking dimers are bound stronger by ca. 4–5 kcal/mol than their crystal structure equivalents. It is interesting to note that the DFT-optimized d3 structures exhibit not only stronger dispersion, but also electrostatic and induction contributions.

It seems paradoxical that the d1, d2 and d4 dimers, relying mostly on electrostatic forces including hydrogen bonds, present more equalized distribution of the interaction energy terms than the stacked d3 dimers (both in the gas phase and in the arrangement from the crystal structure). However, there is another factor which is closely related to the type of force dominating the interactions. Two levels of theory, SAPT0 and SAPT2, are provided in Table 3 and Table 4 to explain this factor. We note that the total SAPT0 and SAPT2 interaction energies are very close to each other when the studied molecules do not engage in hydrogen bonding, highlighting the role of intramonomer electron correlation in the formation of hydrogen bonds. For example, the d3 dimer of **1** has the interaction energy of −13.681 kcal/mol at the SAPT0 level and −13.196 kcal/mol at the SAPT2 level. This means that the hydrogen bonds and electrostatic forces, displaying larger differences between the SAPT0 and SAPT2 energies, contain significant contributions of higher-order corrections connected with electron correlation, not present at the SAPT0 level. On the other hand, the presence of hydrogen bonds in the dimers is associated in this case with a relatively weak interaction (ca. 4–5 kcal/mol). The weakest dimers (d2 type) are rather just multipolar, electrostatic contacts and their particular shape is governed by steric hindrance of the substituents (especially for **2**).

Summarizing the SAPT study, we stress that the stacked arrangement is the principal structural motif of the crystal from the geometric point of view. This fact agrees with the role of dispersion forces as the most important factor from the energetic point of view. However, the details of the solid state structure are modified by the substituents and the polar nature of the compounds introduced not only by the intramolecular hydrogen bonds, but also by the substituents, even relatively mild on the Hammett scale (the methyl groups in **1**, with classical Hammett constants of only −0.07 and −0.17 for $\sigma_{m}$ and $\sigma_{p}$, respectively [63]).

### 2.3. First-Principle Molecular Dynamics (FPMD) in the Gas and Crystalline Phases

The applied Car–Parrinello molecular dynamics (CPMD) enables the investigation of molecular and spectroscopic features of the naphthazarin derivatives based on ab initio Potential Energy Surface (PES), which is of great importance when we are expecting to register proton transfer phenomena events. The time-evolution study provides an insight into the dynamical nature of the hydrogen bonding present in the studied systems. Therefore, special attention was paid to the intramolecular hydrogen bridges present in both compounds. The CPMD simulations were performed in the gas phase and in the solid state. The two phase study enabled detection of differences related to the environmental effects’ influence on the hydrogen bond dynamics, e.g., the crystal field and the presence of neighbouring molecules. The details of the hydrogen bonds’ average metric parameters are presented in Table S2. The reported values are in good agreement with the experimental data available [64,65], as well as the static DFT results.

**Figure 6:**
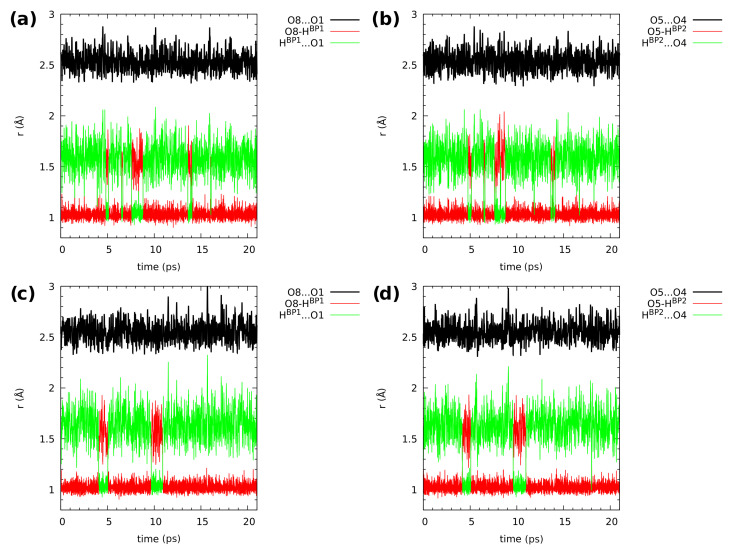
Hydrogen bridge structural parameters during the CPMD simulation of 2,3-dimethylnaphthazarin (**1**). The graphs show gas phase results for (**a**) Bridge 1 and (**b**) Bridge 2, and solid state results for (**c**) Bridge 1 and (**d**) Bridge 2. For atom numbering scheme, see Figure 1.

**Figure 7:**
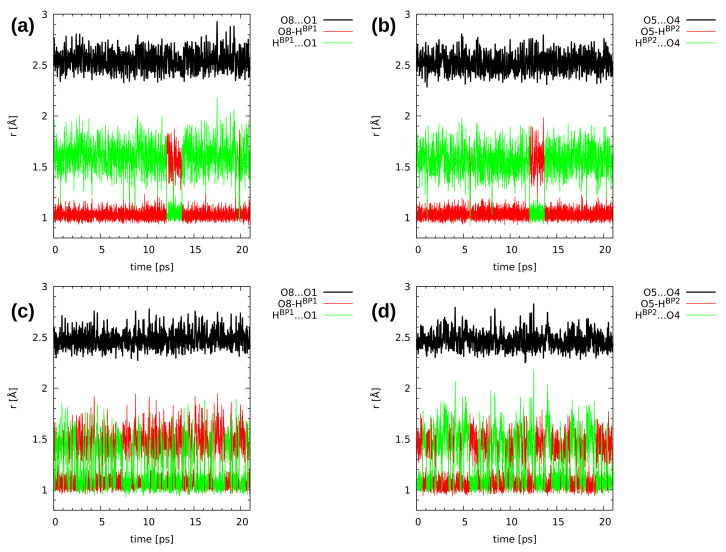
Hydrogen bridge structural parameters during the CPMD simulation of 2,3-dimethoxy-6-methylnaphthazarin (**2**). The graphs show gas phase results for (**a**) Bridge 1 and (**b**) Bridge 2, and solid state results for (**c**) Bridge 1 and (**d**) Bridge 2. For atom numbering scheme, see Figure 1.

Figure 6 and Figure 7, showing the time evolution of the distances related to the hydrogen bridges, use the same color coding to aid data interpretation. The black line corresponds to the O...O donor–acceptor distance, and it simply oscillates around an equilibrium value throughout the simulation time. The red line is the donor-proton bond length, of rather small amplitude, while the green line is the proton-acceptor distance, oscillating in a wide range. The objects of our interest, proton transfer events, are accompanied by a sudden increase in the donor-proton bond length, accompanied by the lowering of the proton-acceptor separation (the red and green lines cross over).

In Figure 6, the hydrogen bridge dynamics is presented for compound **1**. The upper part shows data obtained in the gas phase. There are many proton-sharing events during the CPMD simulation run. The bridged protons exhibit strong mobility, which results in proton transfer phenomena registered during the 20 ps of the CPMD run. The protons moved to the acceptor-atom side, stayed there for a short time and kept moving again to the proton-donor side. In the solid state (lower part of the Figure 6), the proton transfer events were noticed as well. However, there were less proton-sharing events comparing to the gas phase results (see also the discussion of proton possession statistics two paragraphs below). This could be explained by the presence of intermolecular hydrogen bonds and molecular overlapping present in the crystal structure [64]. The presence of neighbouring molecules forming intermolecular hydrogen bonds (O-H...O), where the O-H group is involved in the intramolecular hydrogen bond, as well as interacts with an oxygen atom (proton-acceptor from another molecule) and introduces competition in the interactions. This shows a significant difference between the isolated molecule and the crystalline phase dynamics, where many factors are included during the CPMD simulations. There is a visible correlation in the bridged protons dynamics in both phases. Compound **1** exhibits symmetry; therefore, one could expect that the dynamical nature of the bridged protons will be similar, but it will depend on the phase discussed—the crystal packing lowers the effective symmetry perceived by the analyzed molecule.

The CPMD results concerning the intramolecular hydrogen bond dynamics of compound **2** are presented in Figure 7. The bridged protons exhibit strong mobility in both studied phases. In the gas phase (upper part of Figure 7), there are frequent proton-sharing events and proton transfer phenomena were noticed as well. During the 20 ps run, there were 3 ps long proton transfer events, and after this time, the bridged protons moved back again to the proton-donor atom. There is also a correlation in the bridged protons dynamics—the 3 ps long PT events happened at the same time for both bridges. A solid state study provided a different picture of the proton mobility in the hydrogen bridges (lower part of Figure 7). The bridged protons were strongly delocalized between the donor and acceptor atoms in both hydrogen bonds. The compound did not exhibit symmetry due to the presence of the methyl group in the sixth position as well as methoxy groups, which are not equivalent [65]. There were also intermolecular hydrogen bonds, involving (similarly to compound **1**) OH groups from the intramolecular hydrogen bonds and proton-acceptor atoms from the neighbouring molecules. There was also molecular overlapping according to the X-ray measurements [65]. Comparing gas phase results with the solid state of compound **2**, it is visible from the structural data analysis that external forces influence the bridged protons dynamics. In the case of compound **2**, we can draw the conclusion that the presence of methoxy groups and the lack of symmetry introduced inductive and steric effects, which provided us with a different dynamical nature of the intramolecular hydrogen bonds present in compound **2** with respect to **1**.

The diverse properties of hydrogen bonds were further analyzed using statistics-based approaches. First, we calculated the proton possession statistics, i.e., percentages of the time spent by the given bridge proton at the donor or acceptor site. The proper association of the proton with its site at a given time was determined by the Voronoi geometric criterion—donor-proton vs. proton-acceptor distance comparison. The results, gathered in Table 5, indicate that in case of compound **1**, gas phase and solid state statistics are very similar. The degree of convergence of the dynamics trajectory can be estimated by comparison of the two equivalent bridges, O8...O1 vs. O5...O4—the differences are no more than 0.5%. The differences between the gas phase and solid state results are 0.2% for the O8...O1 bridge and 1.1% for the O5...O4 bridge, which is close to the difference between the two bridges. This means that the solid state environment does not seem to change the overall partitioning of the proton residence time between the donor and the acceptor sites, and it promotes slower dynamics (fewer proton sharing events). The results for the gas phase CPMD simulation of **2** are also similar to the case of **1**: the two bridges, which are not equivalent, are still similar enough to provide the same statistics of proton possession. The solid state case is more interesting: the packing forces (the presence of neighbours and their electrostatic field) lead to almost equally shared protons; however, the H${}^{BP1}$ tends to reside more at the acceptor site than the H${}^{BP2}$ proton.

**Figure 8:**
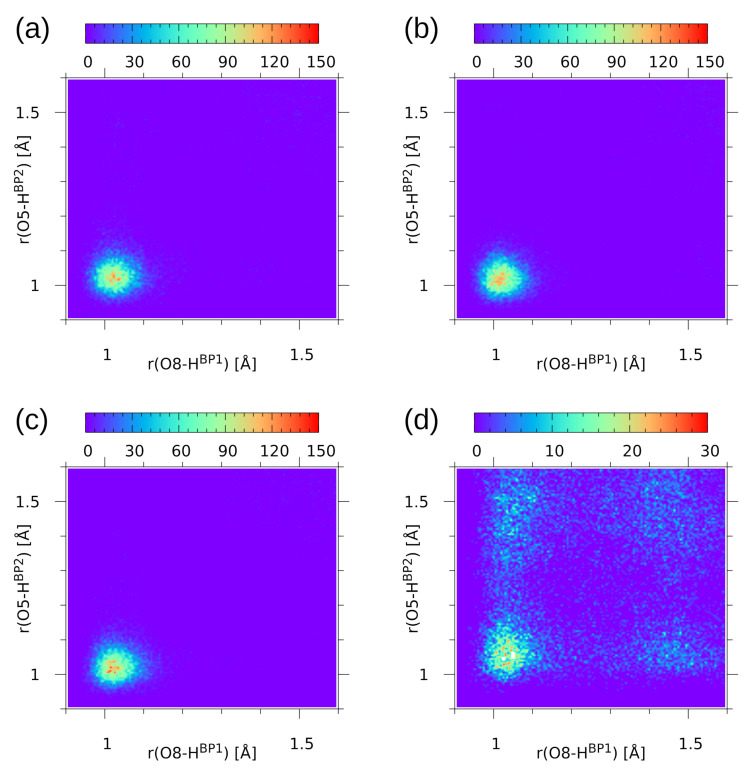
Histograms for the donor-proton distances in the two hydrogen bridges of the studied compounds—results of the CPMD simulation for (**a**) **1** in the gas phase, (**b**) **1** in the solid state, (**c**) **2** in the gas phase, (**d**) **2** in the solid state. Color scale represents probability density in Å${}^{-2}$.

The second part of the CPMD trajectory statistical analysis is provided by the histograms for the donor-proton positions in the two hydrogen bridges—see Figure 8. The histograms (probability density plots) show how the proton positions are correlated in the sense of averaging over the CPMD run. It is visible that for compound **1**, regardless of the simulation conditions—the gas phase or the solid state—the two protons H${}^{BP1}$ and H${}^{BP2}$ are strongly correlated and located mostly at the donor site. This is also true for the gas phase trajectory of **2**. These results are in agreement with the data for naphthazarin [61]: it was shown that when an asynchronous proton jump occurs, it is very probable that a second proton transfer will follow within a few O-H oscillation periods. From this point of view, it is interesting to note that the solid state simulation of **2**, where the protons are more delocalized, also exhibits important motion correlations. The histogram shown in panel (d) of Figure 8 consists of four more populated regions forming a square shape. These regions correspond to the molecular form, the PT form, and the two less stable forms with single-proton transfer. There are no indications of a synchronous double-proton transfer, which would result in formation of a populated region in the center of the square shape.

**Figure 9:**
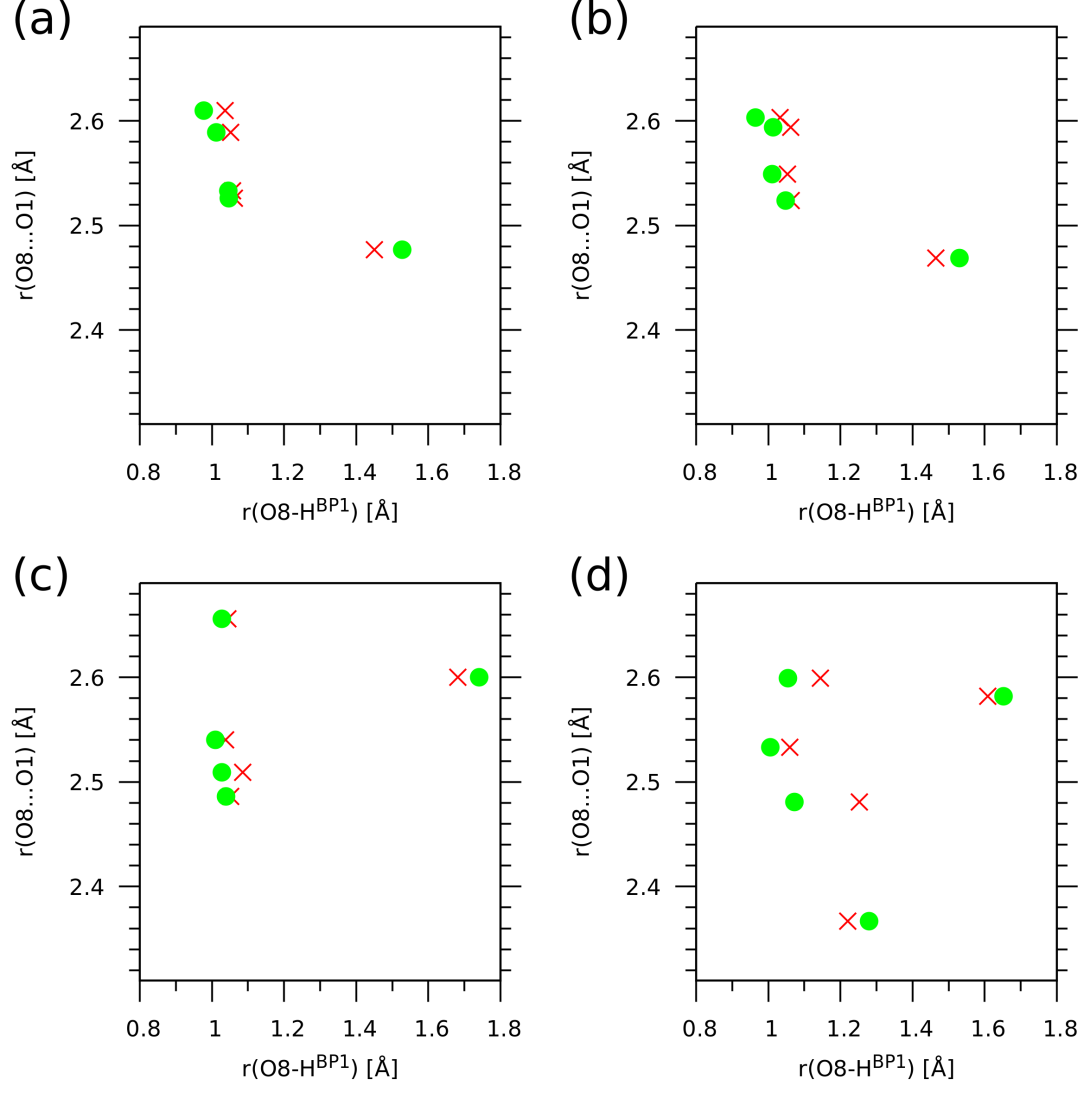
Impact of nuclear quantum effects for the H${}^{BP1}$ bridge proton on the O8-H${}^{BP1}$ distance. Green circles—classical value of the distance; red crosses—quantum expectation value of the O8-H${}^{BP1}$ distance operator. Results of a posteriori quantum treatment of CPMD trajectory for (**a**) **1** in the gas phase, (**b**) **1** in the solid state, (**c**) **2** in the gas phase, (**d**) **2** in the solid state.

While the current study was carried out within the Newtonian classical nuclear dynamics, corresponding to the Born–Oppenheimer picture, it is recognized that in some instances the nuclear quantum effects (NQE) are important for qualitative and quantitative agreement with experiments. An excess proton in water migrates due to a complicated mechanism in which quantum fluctuations, rather than tunneling, play a crucial role [75,76]. Quantum disorder in the hydrogen bonds is required to explain the X-ray absorption spectra of water and ice [77]. Intramolecular hydrogen bonds can be diversely affected by nuclear quantization. Picolinic acid N-oxide with a very strong O-H...O bond requires anharmonic, quantum treatment of the proton motion to rationalize enormous red shifts of the $\nu_{OH}$ mode [78]. Weaker hydrogen bonds, such as those in o-hydroxy Mannich bases, exhibit a single-well potential with the minimum clearly at the donor side [79], while in the N-oxides of Mannich bases the potentials are very flat and broad, allowing the proton to move almost freely within the bridge [45]. The current study contains an assessment of the importance of nuclear quantum effects for the H${}^{BP1}$ proton. The results, shown in Figure 9, are obtained with the snapshot-based a posteriori approach [79,80] involving numerical solution of a vibrational Schrödinger equation [81]. Our attention was focused on the impact of the NQE phenomena on the O8-H${}^{BP1}$ distance. It is visible in Figure 9 that the NQE tend to increase the proton delocalization between the donor and acceptor sites, making the H${}^{BP1}$ atom shift towards the center of the O8...O1 bridge (the red crosses, indicating the NQE-corrected positions, are located closer to the half of the actual O8...O1 distance than are the green circles—classical positions). For each of the four investigated cases, one of the snapshots presents the PT structure, where the O8-H${}^{BP1}$ distance is larger than 1.5 Å. In such cases, the NQE shift the proton position towards lower O8-H${}^{BP1}$ values. The impact of NQE is not decisive for the proton localization in the studied compounds **1** and **2**, with a very interesting exception of the crystalline phase of **2**. The proton at the O8...O1 distances above 2.5 Å behaves in a way similar to the other cases, but at 2.48 Å the impact of NQE is particularly large. The same distance for **1** and gas phase **2** does not lead to such large NQE; therefore, it seems that this is the precise region of bridge length at which the combination of the molecular structure of **2** and the crystal environment make the NQE (including tunneling) very effective. However, when the bridge is compressed even further—to 2.37 Å—the impact of NQE is again very small. The explanation is as follows: at such a short bridge length, the proton potential is already of the flat single-well type, making this bridge temporarily a “low-barrier hydrogen bond” for which the tunneling effects are negligible [75]. As a final remark to the study of NQE, we note that the classical CPMD trajectory is able to sample this region of the molecular phase space, as seen in Figure 8. This fact indicates that the NQE should not have a qualitative impact on the properties of the investigated systems.

**Figure 10:**
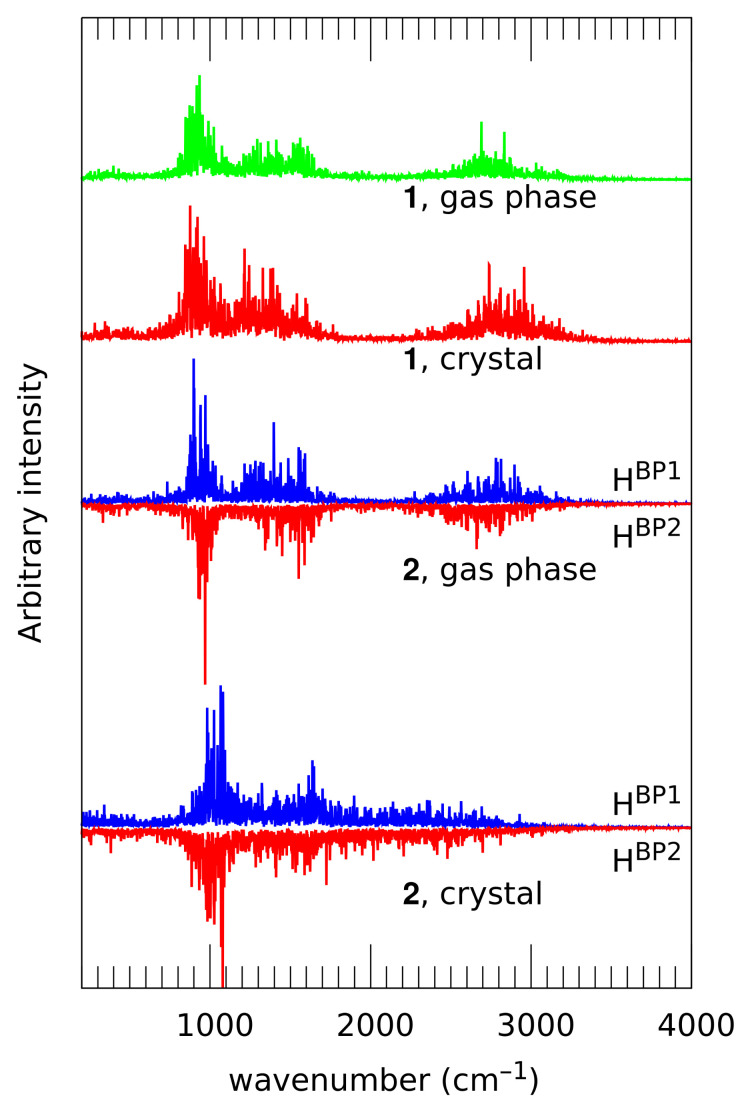
Vibrational signatures (atomic velocity power spectra) of the bridge protons calculated from the CPMD simulation of **1** and **2**. In the case of **2**, signatures of the non-equivalent bridge protons are presented as separate curves placed back to back. For atom numbering scheme, see Figure 1.

Vibrational signatures of the bridged protons, corresponding to the $\nu_{\mathrm{OH}}$ at the high-wavenumber region, are presented in Figure 10. Since the IR spectra of these compounds are not available in the literature, the most natural source of comparison is the parent compound, naphthazarin. Investigation of the bridge proton features has two main goals. First, it is possible to trace the presence of strong interactions in the crystal. On the other hand, the bridges in **2** are not symmetrical, and their asymmetry can lead to slightly diverse positions of the normal modes. This is an interesting issue in relation to the parent naphthazarin itself, where the skeleton, devoid of the substituents, does not prefer any of the proton positions. The broad absorptions of the νOH/OD and γOH/OD stretching modes were experimentally identified in naphthazarin at 3060/2200 cm${}^{-1}$ and 793/560 cm${}^{-1}$, respectively; the upper wavenumber region is the most relevant for the fast proton dynamics corresponding to the stretching mode [62].

The first goal, detection of strong interactions, can be accomplished by comparison of the $\nu_{\mathrm{OH}}$ band positions. Compound **1** exhibits similar positions of this band in the gas phase (from 2300 to 3400 cm${}^{-1}$) and crystal (from 2200 to 3400 cm${}^{-1}$). The band center at ca. 2800 cm${}^{-1}$–2900 cm${}^{-1}$ is at a slightly lower wavenumber than the experimental value of 3060 cm${}^{-1}$ for naphthazarin [62]. The lower wavenumber absorptions, 700–1700 cm${}^{-1}$ in the gas phase and 600–1800 cm${}^{-1}$ in the solid state, should be attributed to the mechanical influence of the heavy-atom motions. These values indicate on the one hand a middle-strong O-H...O hydrogen bonding, and on the other a relatively small impact of the crystal packing effects on the vibrational features of **1**. These facts agree well with the not too frequent proton transfer events in this compound (see Figure 6, which also confirms that the PT occurrence in the gas phase and in the crystal is very similar). We have noted already that the PT events are not strictly synchronous, but they are strongly correlated. This makes the vibrational signatures of H${}^{BP1}$ and H${}^{BP2}$ virtually identical. This is not strictly true for compound **2**, where the chemical nature of the substituents is different in the vicinity of H${}^{BP1}$ than in the vicinity of H${}^{BP2}$. The difference is almost not visible in the results of the gas phase simulation of **1**—the $\nu_{\mathrm{OH}}$ vibrational features of both protons fall into the 2300 cm${}^{-1}$ to 3300 cm${}^{-1}$ range (the 700 cm${}^{-1}$–1700 cm${}^{-1}$ region is associated with heavy atom motions, as already noted for compound **1**), and the signature of H${}^{BP1}$ is centered at ca. 2800 cm${}^{-1}$, while the signature of the H${}^{BP2}$ proton peaks at ca. 100 cm${}^{-1}$ has a lower wavenumber. The difference is small, and it is also in agreement with the time evolution of the distance parameters (see Figure 7). The lowering of the band center position with respect to naphthazarin (3060 cm${}^{-1}$ in the experimental spectrum) is also not large. Quite unexpectedly (if one has not yet appreciated the solid state distance parameters shown in Figure 7), the crystal field makes the bridge protons very strongly delocalized. The resulting vibrational signature is extremely broad and forms a continuous background feature from ca. 700 to 3100 cm${}^{-1}$. This feature does not differentiate the two bridge protons. The reason for such a profound change in the bridge proton dynamics should be sought after for a particular arrangement of molecules in crystal; thus, the competition between inter- and intramolecular contacts turns out to be cooperation in the case of the solid state compound, compound **2**.

## 3. Computational Methodology

### 3.1. Static Models on the Basis of Density Functional Theory (DFT)

The models of monomers and dimers were constructed on the basis of X-ray structures of 2,3-dimethylnaphthazarin (**1**) (CCDC deposition number—1125030) and 2,3-dimethoxy-6-methylnaphthazarin (**2**) (CCDC deposition number—1161869) [64,65,82]. The geometry optimization for the molecular forms of monomers was performed using Density Functional Theory (DFT) [66,67] and three functionals: B3LYP [83], PBE [84,85] and ωB97XD [86] with valence-split triple-zeta Pople’s style basis set denoted as 6-311++G(2d,2p) [87,88]. The choice of functionals was devised to represent the current spectrum of the most widely used approaches: the PBE functional is of the Generalized Gradient Approximation (GGA) type used frequently in the context of plane-wave calculations (including Car–Parrinello MD), and does not use the exact exchange. On the other hand, B3LYP is a hybrid functional, and so is the ωB97XD, but the latter includes empirical dispersion correction. Following the geometry optimization, harmonic frequencies were computed to confirm that the obtained structures correspond with the minimum on the Potential Energy Surface (PES). Additionally, models with diverse proton positions were constructed and optimized as well using the DFT method (for details, see Table S1 of the Supplementary Information). In the next step, the single-point simulations at the MP2 [89] and CCSD [90,91] levels with def2-TZVP basis set [92] were carried out for the structures of the minima and transition state on the PT pathway. Next, the structures with OH groups on the proton-donor side were taken to investigate the proton potential paths using the scan method with geometry optimization (the O-H increment was set to 0.05 Å, the O8H${}^{BP1}$O1 and O5H${}^{BP2}$O4 valence angles were frozen while the remaining parts of the molecules were optimized). The results of the scans formed a discrete set of points, from which a proton potential function was derived. Thus, the barrier height is determined with accuracy depending on the discrete steps of energy in the vicinity of the transition state; the error estimate is the internal property of the procedure based on the discrete series of points, not the absolute uncertainty of a particular DFT functional. The zero-point vibrational correction is not included in the reported values. Finally, the wavefunctions for the Atoms In Molecules (AIM) theory [69] analysis were prepared with assistance of the B3LYP functional and 6-311++G(2d,2p) basis set for molecular and proton transferred forms of monomers. The theory was applied for the electronic structure as well as molecular topology investigations. Special attention was paid to the electron density and its Laplacian values at Bond and Ring Critical Points (BCPs and RCPs) related to the intramolecular hydrogen bonding. Next, for the dimeric structures extracted from the crystal data of compounds **1** and **2** [64,65], the energy minimization was performed using the ωB97XD functional [86] and 6-311++G(2d,2p) basis set. The simulations were carried out in the gas phase with the Gaussian 09 rev. D.01 [93] and Gaussian 16 rev. C.01. suite of programs [94]. The single-point MP2 and CCSD calculations were conducted with the Turbomole 6.5 program [95]. The AIM analysis was performed using the AIMAll program [96]. In addition, sets of coordinates are provided in the Supplementary Information for the current study.

### 3.2. An Application of Symmetry-Adapted Perturbation Theory (SAPT) to Dimers

The Symmetry-Adapted Perturbation Theory (SAPT) [70] enables energy decomposition between interacting molecules, in our case dimers. The method divides an exact Hamiltonian into Hartree–Fock contribution of monomers, ${\hat{F}}_{A}$ and ${\hat{F}}_{B}$, correlation components interacting inside the monomers, ${\hat{W}}_{A}$ and ${\hat{W}}_{B}$, and the contribution covering interaction between monomers, $\hat{V}$:

(1) $$\hat{H}={\hat{F}}_{A}+{\hat{F}}_{B}+{\hat{W}}_{A}+{\hat{W}}_{B}+\hat{V}$$

An important advantage of the SAPT scheme is the fact the individual components could be grouped into four principal groups with precisely defined physical interpretation: (i) electrostatic ($E_{elst}$)—approximate Coulombic interactions of electron density decomposition of isolated monomers (without the effect of polarization by the neighboring molecule); (ii) exchange ($E_{exch}$—which is the short-range Pauli repulsion; (iii) Induction ($E_{ind}$) and exchange-induction ($E_{ex-ind}$—which is based on mutual polarization of the monomers; (iv) dispersion ($E_{disp}$)—consideration of short-lived instantaneous multipoles. Depending on the considered energy components, the SAPT hierarchy of interactions is obtained. The SAPT levels most commonly used are SAPT0 (in agreement with Hartree–Fock method) and SAPT2 (with accuracy approximate to the MP2 method):

(2) $$E_{SAPT0}=E_{elst}^{10}+E_{exch}^{10}+E_{ind,r}^{20}+E_{ex-ind,r}^{20}+\delta E^{HF}+E_{disp}^{20}+E_{ex-disp}^{20}$$

(3) $$E_{SAPT2}=E_{SAPT0}+E_{elst,r}^{12}+E_{exch}^{11}+E_{exch}^{12}{+}^{t}E_{ind}^{22}{+}^{t}E_{ex-ind}^{22}$$

These equations show the fundamental difference between the SAPT0 and SAPT2 approximations: the SAPT0 components never use intramonomer electron correlation, so—generally speaking—the resulting components of interaction energy are based on the non-correlated Hartree–Fock wavefunctions of the monomers. SAPT2, on the other hand, includes intramonomer correlation up to the second perturbative order, which is especially important for very weak interactions. In our experience with hydrogen-bonded systems, SAPT0 results are overestimated in comparison to the more accurate SAPT2 approach, but the general trends are reproduced with quite a high degree of correlation between the methods. Regarding the computational efficiency and memory requirements, SAPT2 can be prohibitively demanding for systems of ca. 60 atoms. However, due to the electron density expansion on specially fitted basis functions (density fitting technique), the SAPT0 computational cost is comparable to the MP2 method.

The energy decomposition of the naphthazarin derivative dimers (see Figure 5) was performed for: (i) data extracted from the X-ray structures of the investigated compounds [64,65] in order to reproduce the intermolecular forces in the crystal structure responsible for the crystal unit cell arrangement; (ii) the data obtained as a result of gas phase DFT simulations at the ωB97XD/6-311++G(2d,2p) level of theory. The interaction energy was calculated at the SAPT2/jun-cc-pVDZ level of theory (truncation of the diffuse functions in the jun-cc-pVDZ basis is derived in [97]). The basis set superposition error (BSSE) correction [98] was included in the simulations of the dimers (the studied dimers were divided into “monomers” in order to fulfil the requirements of the Boys–Bernardi method). The SAPT calculations were carried out using the Psi4 1.2.1 [99] program.

### 3.3. Car-Parrinello Molecular Dynamics in the Gas Phase and Solid State

The dynamical nature of the studied naphthazarin derivatives (compounds denoted as **1** and **2**, see Figure 1 and Figure S2) [64,65] were examined in the light of First-Principle Molecular Dynamics (FPMD) method. The simulations were performed for the isolated molecules as well as for the molecular crystals. The gas phase simulations results were further used for the comparative study of differences introduced by the interatomic forces present in the solid state. Our attention was placed on the intramolecular hydrogen bonds’ dynamics and properties. We have analyzed the hydrogen bridges dynamics as a function of simulation time. For this purpose, detailed analysis of metric parameters was performed for O1...O8/O5...O4 interatomic distance, O1-H${}^{BP1}$/O2-H${}^{BP2}$ covalent bonds and H${}^{BP1}$...O8/H${}^{BP2}$...O4 intramolecular hydrogen bonds in compound **1**. Compound **1** is symmetric; therefore, we could expect that the bridged proton dynamics will be similar. However, we placed emphasis on a detailed view of protons motion in the hydrogen bridges. Compound **2** has a broken symmetry due to the presence of the CH${}_{3}$ substituent. Both hydrogen bridges were taken into consideration in the analysis of metric parameters. We were looking for any correlations in the hydrogen bridge dynamics. Another aspect related to the data analyses were vibrational signatures provided by the OH groups. The Fourier transformation of the autocorrelation function of atomic velocity was employed to develop power spectra. The models used for Car–Parrinello molecular dynamics (CPMD) in the gas phase are presented in Figure 1. The initial geometries for the isolated molecules were extracted from the X-ray data [64,65] and placed in cubic boxes with a = 15 Å for compound **1** and a= 16 Å for compound **2**. The models for CPMD in the solid state were prepared on the basis of crystallographic unit cells [64,65]. The unit cell dimensions for compound **1** are as follows: a = 16.429 Å, b = 6.524 Å, c = 9.136 Å and β = 90.19${}^{\circ }$ with Z = 4, while for compound **2**, a = 3.873 Å, b = 20.21 Å, c = 15.00 Å and β = 96.05${}^{\circ }$ with Z = 4. The computational setup for the simulations in both studied phases was prepared bearing in mind the fact that intramolecular hydrogen bond dynamics were being studied. The simulations were divided into geometry optimization of the studied compounds, **1** and **2**, and subsequent CPMD runs in the gas phase and solid state. The exchange correlation functional by Perdew, Burke and Ernzerhof (PBE) [84,85] and Troullier–Martins [100] pseudopotentials were applied. The fictitious electron mass (EMASS) was equal to 400 a.u. and the time-step was set to 3 a.u. The kinetic energy cutoff for the plane-wave basis set was 80 Ry. The CPMD calculations were performed at 295 K, controlled by Nosé–Hoover thermostat chain assigned to ions [101,102]; the electronic system was thermostatted at the orbital kinetic energy values determined in separate short non-thermostatted runs for each system. Hockney’s scheme [103] was applied to remove interactions with periodic images of the cubic cell during the gas phase dynamics. The translational and rotational movements were removed from the CPMD data collection as well. The crystalline phase CPMD was carried out with Γ point approximation [104] and Periodic Boundary Conditions (PBCs). The real-space electrostatic summation was set to TESR = 8 nearest neighbours in each direction. The CPMD simulations were divided into two parts: (i) equilibration (the initial part of the trajectory—ca. 10,000 steps—was removed from further analyses); (ii) production run, which lasted for 21 ps.

The CPMD simulations were performed using the CPMD 3.17.1 program [105]. The post-processing was carried out using home-made scripts and the VMD 1.9.3 [106] program. The graphical presentation of the obtained results in the current study was conducted with assistance of the VMD 1.9.3 [106] and Gnuplot [107] programs.

### 3.4. Estimation of the Nuclear Quantum Effects on the Structural Properties in the Gas Phase and Solid State

The nuclear quantum effects for the bridge proton motion were studied using an a posteriori approach based on the CPMD trajectory [79,80]. In short, the method consists of selecting several snapshots from the CPMD trajectory, calculating proton potential functions for each snapshots, and then, finally, solving the vibrational Schrödinger equation (see, e.g., [79,81]). The particular details for the current study are as follows. Four cases were considered: compounds **1** and **2** in the gas phase and solid state. For each case, five snapshots were extracted from the CPMD trajectory with constant time intervals. For each snapshot, a set of 16 to 20 bridge proton positions (depending on the donor–acceptor distance) was generated for the scan using the donor, proton and acceptor coordinates to define a fragment of an arc. The generated proton positions were then used to calculate single-point energies for the studied systems using the corresponding computational setup of the CPMD code—see the section above. Then, each of the generated proton potential profiles was fitted with a 9^th^ degree polynomial, and a one-dimensional vibrational Schrödinger equation was solved using a grid basis set of 400 points spanning the O8-H${}^{BP1}$ region from 0.7 Å to 2.0 Å. Finally, the expectation value of the O8-H${}^{BP1}$ distance operator at 295 K was calculated taking into account the three lowest-lying vibrational levels. The electronic structure calculations were carried out with the CPMD 3.17.1 program [105], while the quantum vibrational effects were studied with the software developed by Stare and Mavri [81].

## 4. Conclusions

We have presented computational results of two naphthazarin derivatives substituted with methyl and methoxy groups in diverse manner. We have examined various factors influencing the molecular features exhibited by the aforementioned derivatives in relation to the properties of the substituents and symmetry breaking by their introduction. The presence of the substituents and changes in the chemical composition have led to changes in the bridged proton dynamics and intermolecular interactions in comparison to the parent compound, naphthtazarin. The computations were performed in the electronic ground state, both in the gas phase and solid state. In order to shed light on the intermolecular interactions, the dimers of compounds **1** and **2** were investigated. Our computational findings were compared with the experimental data available (structural and spectroscopic). The application of the DFT method with three different functionals, each using a 6-311++G(2d,2p) basis set, complemented with the single-point MP2 and CCSD calculations with the def2-TZVP basis set, provided information of the proton reaction path and the energy barrier for the proton transfer. The highest DFT energy barrier equals ca. 10 kcal/mol, while MP2 and CCSD provided the barrier heights of ca. 6.8 and 9.6 kcal/mol, respectively. Moreover, two energy minima were located in both molecules and in both examined hydrogen bridges. The application of the AIM theory gave a quantitative picture of the electron density distribution in the molecular and proton transferred forms of the studied compounds. The topological analysis confirmed the presence of the intramolecular hydrogen bonds (in agreement with experimental X-ray findings in the literature). Additionally, it was shown, on the basis of electron density and its Laplacian values, that the hydrogen bonds are stronger in the tautomeric PT forms. The SAPT analysis gave an insight into energy partitioning and provided information on the primary factors responsible for dimer stabilization. It was found that the primary factors are the dispersive forces. Using the SAPT method, we could identify and describe quantitatively external forces influencing the molecular features of compounds **1** and **2**. The CPMD results showed that protons in the hydrogen bridges are very labile. Proton transfer phenomena were observed in the gas phase as well as in the solid state. In compound **2**, there is a clearly visible influence of environmental factors on the hydrogen bridge dynamics. The vibrational analysis confirmed, by the broad absorption regions observed in the computed power spectra, a strong anharmonicity of the studied hydrogen bonds as well as their dynamics. It is especially visible in compound **2**, where in the solid state only one very broad absorption (700 cm${}^{-1}$–3100 cm${}^{-1}$) region was found. The incorporation of nuclear quantum effects to the hydrogen bridges showed a stronger delocalization of the bridged protons, especially at shorter, but not the shortest, distances between the donor and acceptor heavy atoms. 

## Figures and Tables

**Table 1:** Atoms In Molecules (AIM) atomic charges calculated for selected atoms of the studied compounds, compounds **1** and **2**, and their proton transferred (PT) forms at the B3LYP/6-311++G(2d,2p) level of theory.

Atomic Charge [e]	Compound 1		Compound 2	
	Molecular Form	PT Form	Molecular Form	PT Form
	**Hydrogen Bridge 1**			
O8	−1.134	−1.094	−1.134	−1.097
$H^{BP1}$	0.641	0.643	0.641	0.642
O1	−1.099	−1.139	−1.081	−1.119
C8	0.597	0.874	0.597	0.874
C9	−0.027	−0.030	−0.029	−0.027
C1	0.869	0.587	0.902	0.618
	**Hydrogen Bridge 2**			
O5	−1.134	−1.094	−1.139	−1.104
$H^{BP2}$	0.641	0.642	0.642	0.645
O4	−1.098	−1.137	−1.102	−1.136
C5	0.596	0.875	0.582	0.857
C10	−0.028	−0.030	−0.027	−0.029
C4	0.868	0.585	0.873	0.601

**Table 2:** Atoms In Molecules (AIM) Bond Critical Point properties calculated for selected bonds of the studied compounds, compounds **1** and **2**, and their proton transferred forms (PT) at the B3LYP/6-311++G(2d,2p) level of theory. Electron density $\rho_{BCP}$ is given in $e\cdot a_{0}^{-3}$ atomic units, and its Laplacian $\nabla^{2}\rho_{BCP}$ is given in $e\cdot a_{0}^{-5}$ units.

	Compound 1		Compound 2	
BCP	$\rho_{\mathrm{BCP}}$	$\nabla^{2}\rho_{\mathrm{BCP}}$	$\rho_{\mathrm{BCP}}$	$\nabla^{2}\rho_{\mathrm{BCP}}$
	Molecular Form			
O8-$H^{BP1}$	0.339	−2.536	0.340	−2.540
$H^{BP1}$-O1	0.051	0.138	0.050	0.136
O5-$H^{BP2}$	0.339	−2.533	0.337	−2.511
$H^{BP2}$-O4	0.051	0.137	0.053	0.140
	**Proton-Transferred Form (PT)**			
O8-$H^{BP1}$	0.054	0.139	0.053	0.137
$H^{BP1}$-O1	0.335	−2.487	0.335	−2.488
O5-$H^{BP2}$	0.052	0.137	0.057	0.141
$H^{BP2}$-O4	0.336	−2.505	0.330	−2.441

**Table 3:** SAPT2/jun-cc-pVDZ results of energy partitioning for the dimers of compounds **1** and **2** (see Figure 5) with structures taken directly from the diffraction experiments. All energy terms in kcal/mol: Elst—electrostatics; Exch—exchange (Pauli) repulsion; Ind—induction (polarization); Disp—dispersion; SAPT0 and SAPT2 are defined according to Ref. [74].

Compound	Dimer	Elst	Exch	Ind	Disp	SAPT0	SAPT2
**1**	d1	−3.617	4.754	−0.676	−3.739	−3.342	−3.278
**1**	d2	−0.710	1.716	−0.181	−2.104	−1.328	−1.279
**1**	d3	−3.210	7.190	−0.884	−11.991	−9.233	−8.894
**2**	d1	−4.426	3.857	−0.760	−4.178	−6.305	−5.507
**2**	d2	−0.750	1.598	−0.349	−2.084	−1.601	−1.585
**2**	d3	−6.162	15.081	−2.063	−21.160	−14.795	−14.304
**2**	d4	−5.099	3.273	−1.069	−2.838	−6.849	−5.733

**Table 4:** SAPT2/jun-cc-pVDZ results of energy partitioning for the dimers of compounds **1** and **2** (see Figure 5) with structures taken from the DFT structural optimization. All energy terms in kcal/mol: Elst—electrostatics; Exch—exchange (Pauli) repulsion; Ind—induction (polarization); Disp—dispersion; SAPT0 and SAPT2 are defined according to Ref. [74].

Compound	Dimer	Elst	Exch	Ind	Disp	SAPT0	SAPT2
**1**	d1	−6.116	7.711	−1.112	−4.766	−5.410	−4.283
**1**	d3	−9.129	20.285	−2.485	−21.867	−13.681	−13.196
**2**	d1	−5.830	8.537	−1.187	−5.958	−5.443	−4.438
**2**	d3	−14.669	29.178	−3.754	−29.914	−19.906	−19.158
**2**	d4	−7.830	8.427	−2.254	−4.258	−7.290	−5.916

**Table 5:** Proton possession statistics for the CPMD runs. Percentages of the time spent by the bridge proton at the donor or acceptor site, determined by Voronoi geometric criterion–distance comparison.

O8-H${}^{\mathrm{BP}1}$...O1		O5-H${}^{\mathrm{BP}2}$...O4	
**1**, gas phase			
O8 donor	O1 acceptor	O5 donor	O4 acceptor
89.7%	10.3%	90.2%	9.8%
**1**, solid state			
O8 donor	O1 acceptor	O5 donor	O4 acceptor
89.5%	10.5%	89.1%	10.9%
**2**, gas phase			
O8 donor	O1 acceptor	O5 donor	O4 acceptor
91.6%	8.4%	91.8%	8.2%
**2**, solid state			
O8 donor	O1 acceptor	O5 donor	O4 acceptor
41.6%	58.4%	53.1%	46.9%

## Data Availability

All the relevant processed data (energy values, AIM data, SAPT energy terms, structural information, time evolution of distances in the hydrogen bridges, vibrational signatures) are reported within the manuscript.

## Supplementary Materials

The following are available online. Figure S1. The structures of the investigated naphthazarin derivatives: 2,3-dimethylnaphthazarin (**1**) and 2,3-dimethoxy-6-methylnaphthazarin (**2**), with atom numbering scheme for hydrogen bridges. Coloring scheme: oxygen atom—red, carbon atom—grey and hydrogen atom—white. Figure S2. The models for gas phase and solid state CPMD simulations. Left—the isolated molecule model of 2,3-dimethylnaphthazarin (**1**); right—the model used for solid state simulations of 2,3-dimethoxy-6-methylnaphthazarin (**2**). Table S1. Energy for compounds **1** and **2** with different proton positions in the hydrogen bridges computed using DFT method. Electronic as well as vibrational zero point-corrected values are given. Table S2. Selected geometric parameters related to the intramolecular hydrogen bonds of 2,3-dimethylnaphthazarin (**1**) and 2,3-dimethoxy-6-methylnaphthazarin (**2**). Comparison of experimental and computed data. Metric parameters are given in Å and degrees. CPMD results are presented as average ± standard deviation. Sets of coordinates for the minima and transition state estimates from the DFT scans (XYZ format).

## Abbreviations

The following abbreviations are used in this manuscript:

DFT	Density Functional Theory
CPMD	Car–Parrinello Molecular Dynamics
PT	Proton Transfer
AIM	Atoms In Molecules
SAPT	Symmetry-Adapted Perturbation Theory
NQE	Nuclear Quantum Effects
RAHB	Resonance-Assisted Hydrogen Bond
PES	Potential Energy Surface
MP2	Møller–Plesset second-order perturbation theory
CCSD	Coupled Clusters with Singles and Doubles
BCP	Bond Critical Point
RCP	Ring Critical Point
BSSE	Basis Set Superposition Error
FPMD	First-Principle Molecular Dynamics
PBCs	Periodic Boundary Conditions
