# Supplementary material for: Naphthazarin Derivatives in the Light of Intra- and Intermolecular Forces
Source: Molecules. 2021 Sep 17;26(18):5642. doi: 10.3390/molecules26185642 (PMC8468954; doi:10.3390/molecules26185642)
Supplement: Supplementary file 1 [file molecules-26-05642-s001.zip › molecules-1317190-supplementary.pdf]

SUPPLEMENTARY INFORMATION

Naphthazarin derivatives in the light of intra- and intermolecular forces

Karol Kułacz[1†], Michał Pocheć[1†], Aneta Jezierska[1*], Jarosław J. Panek[1*]

[1] University of Wrocław, Faculty of Chemistry, ul. F. Joliot-Curie 14, 50-383 Wrocław, Poland

[†] These authors contributed equally to this work

Table of content:

I. **Figure S1.** The structures of the investigated naphthazarin derivatives: 2,3-dimethylnaphthazarin (**1**) and 2,3-dimethoxy-6-methylnaphthazarin (**2**) with atoms numbering scheme for hydrogen bridges. Coloring scheme: oxygen atom – red, carbon atom – grey, and hydrogen atom – white.

II. **Figure S2.** The models for gas phase and solid state CPMD simulations. Left – the isolated molecule model of 2,3-dimethylnaphthazarin (**1**); right – the model used for solid-state simulations of 2,3-dimethoxy-6-methylnaphthazarin (**2**).

III. **Table S1.** Energy for compounds **1** and **2** with different proton positions in the hydrogen bridges computed using DFT method. Electronic as well as vibrational zero point-corrected values are given.

IV. **Table S2.** Selected geometric parameters related to the intramolecular hydrogen bonds of 2,3-dimethylnaphthazarin (**1**) and 2,3-dimethoxy-6-methylnaphthazarin (**2**). Comparison of experimental [1, 2] and computed data. Metric parameters are given in Å and degrees. CPMD results are presented as average ± standard deviation. For atoms numbering scheme see Figure S1.

V. **Sets of coordinates** for the minima and transition state estimates from the DFT scans (XYZ format).

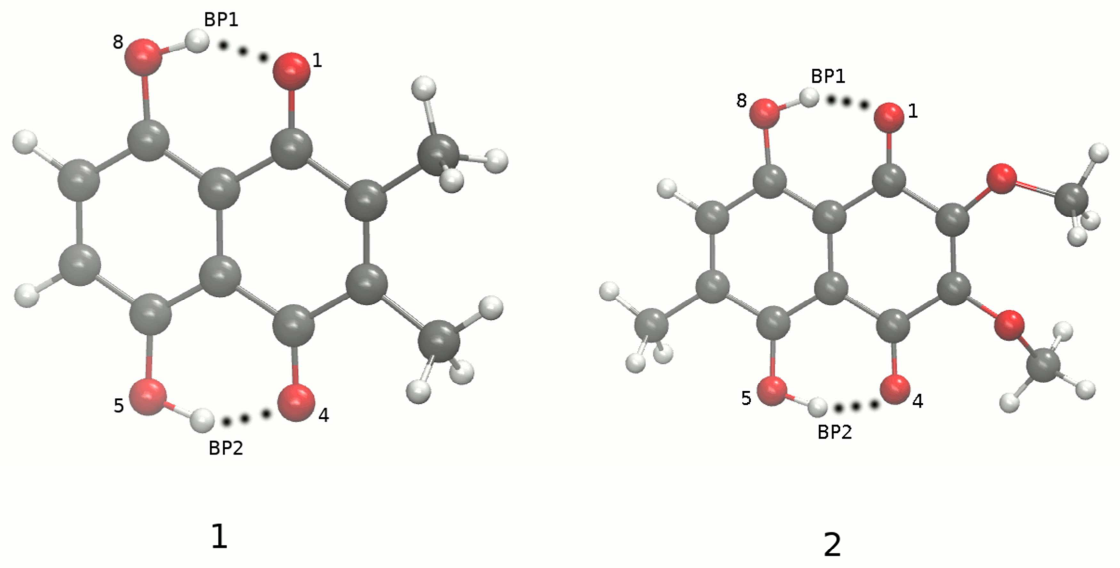

Figure S1. The structures of the investigated naphthazarin derivatives: 2,3-dimethylnaphthazarin (**1**) and 2,3-dimethoxy-6-methylnaphthazarin (**2**) with atoms numbering scheme for hydrogen bridges. Coloring scheme: oxygen atom – red, carbon atom – grey and hydrogen atom – white.

O8-H^{BP1}…O1 – Bridge 1
O5-H^{BP2}…O4 – Bridge 2

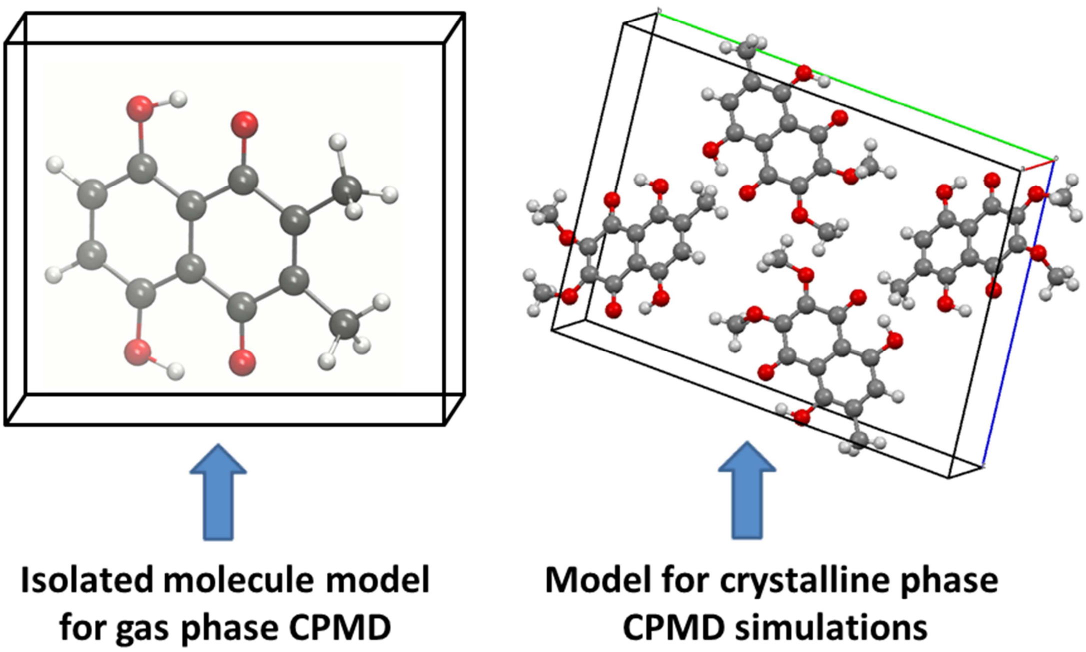

Figure S2. The models for gas phase and solid state CPMD simulations. Left – the isolated molecule model of 2,3-dimethylnaphthazarin (**1**); right – the model used for solid-state simulations of 2,3-dimethoxy-6-methylnaphthazarin (**2**).

Table S1. Energy for compounds **1** and **2** with different proton positions in the hydrogen bridges computed using DFT method. Electronic as well as vibrational zero point-corrected values are given.

**Compound 1 – electronic energy**		
**Molecular form**	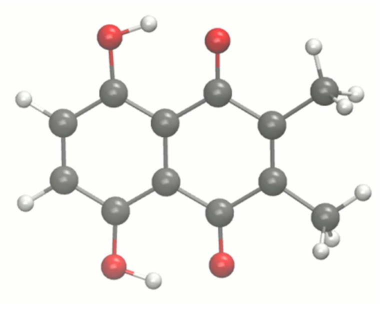	**Energy [kcal/mol]**<br>**B3LYP = -479702.5025**<br>**PBE = -479148.6994**<br>**ωB97XD = -479538.1104**
**Proton on the donor side in the Bridge 1 and proton on the acceptor side in the Bridge 2**	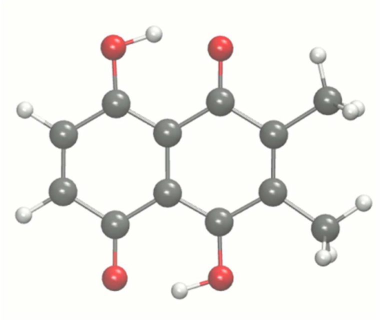	**B3LYP = -479697.1997**<br>**PBE = -479145.8773**<br>**ωB97XD = -479531.296**
**Proton-transferred form**	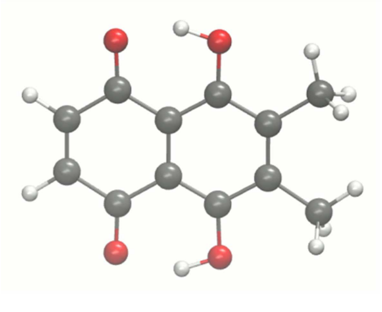	**B3LYP = -479700.5659**<br>**PBE = -479147.0874**<br>**ωB97XD = -479536.0907**

*** Basis set used for the simulations: 6-311++G(2d,2p)**

***O8-H^{BP1}…O1 – Bridge 1**

O5-H^{BP2}…O4 – Bridge 2

Table S1 Continuation. Energy for compounds **1** and **2** with different proton positions in the hydrogen bridges computed using DFT method. Electronic as well as vibrational zero point-corrected values are given.

**Compound 1 – electronic energy with correction for vibrational zero point energy (ZPE) included**		
**Molecular form**	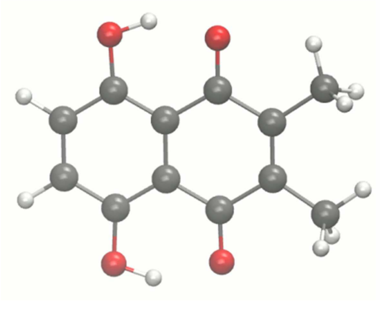	**Energy+ZPE [kcal/mol]**<br>**B3LYP = -479578.7114**<br>**PBE = -479028.9699**<br>**ωB97XD = -479412.1866**
**Proton on the donor side in the Bridge 1 and proton on the acceptor side in the Bridge 2**	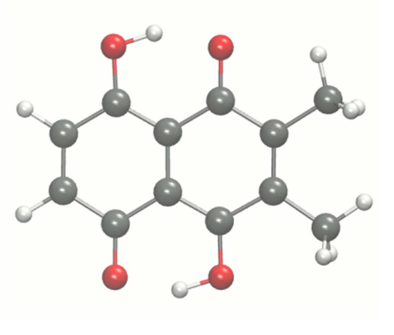	**B3LYP = -479574.0566**<br>**PBE = -479027.7105**<br>**ωB97XD = -479405.9611**
**Proton-transferred form**	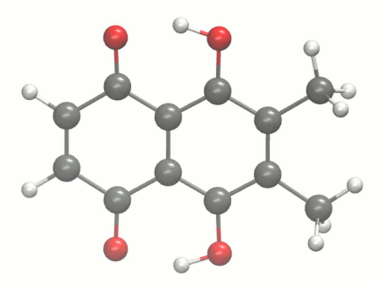	**B3LYP = -479576.8051**<br>**PBE = -479027.5938**<br>**ωB97XD = -479410.6442**

*** Basis set used for the simulations: 6-311++G(2d,2p)**

***O8-H^{BP1}…O1 – Bridge 1**

O5-H^{BP2}…O4 – Bridge 2

Table S1 Continuation. Energy for compounds **1** and **2** with different proton positions in the hydrogen bridges computed using DFT method. Electronic as well as vibrational zero point-corrected values are given.

**Compound 2 – electronic energy**		
		**Energy [kcal/mol]**
**Molecular form**	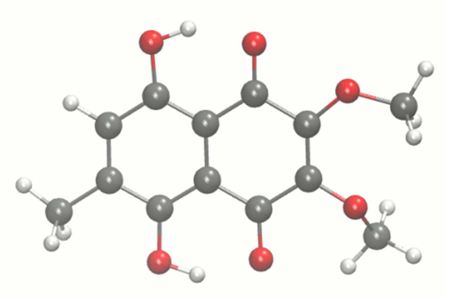	**B3LYP = -598790.8323**<br>**PBE = -598112.0786**<br>**ωB97XD = -598588.4445**
**Proton on the acceptor side in the Bridge 1 and proton on the donor side in the Bridge 2**	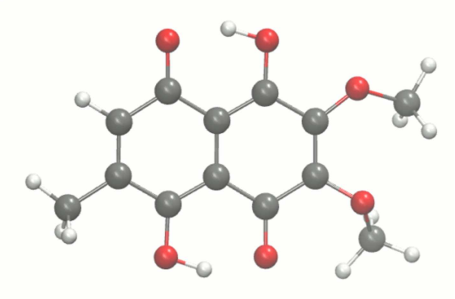	**B3LYP = -598785.446**<br>***PBE = -598112.0787**<br>**ωB97XD = -598581.7599**
**Proton on the donor side in the Bridge 1 and proton on the acceptor side in the Bridge 2**	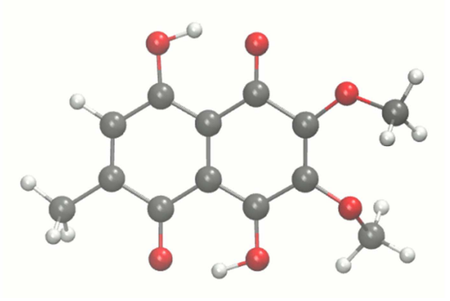	**B3LYP = -598785.2588**<br>***PBE = -598112.0787**<br>**ωB97XD = -598581.4126**
**Proton-transferred form**	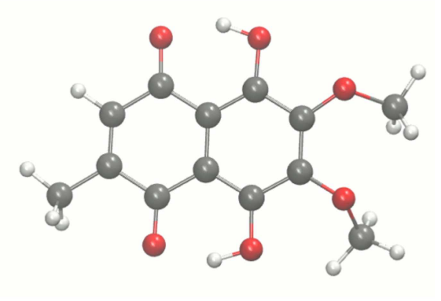	**B3LYP = -598787.8919**<br>**PBE = -598109.3002**<br>**ωB97XD = -598585.5316**

*** Basis set used for the simulations: 6-311++G(2d,2p)**

***O8-H^{BP1}…O1 – Bridge 1**

O5-H^{BP2}…O4 – Bridge 2

*** in case of PBE functional the molecular form was obtained**

Table S1 Continuation. Energy for compounds **1** and **2** with different proton positions in the hydrogen bridges computed using DFT method. Electronic as well as vibrational zero point-corrected values are given.

**Compound 2 – electronic energy with correction for vibrational zero point energy (ZPE) included**		
		**Energy+ZPE [kcal/mol]**
**Molecular form**	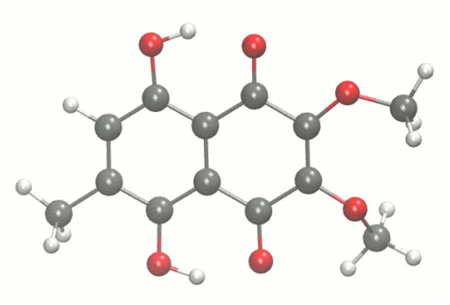	**B3LYP = -598643.6520**<br>**PBE = -597969.6629**<br>**ωB97XD = -598438.8419**
**Proton on the acceptor side in the Bridge 1 and proton on the donor side in the Bridge 2**	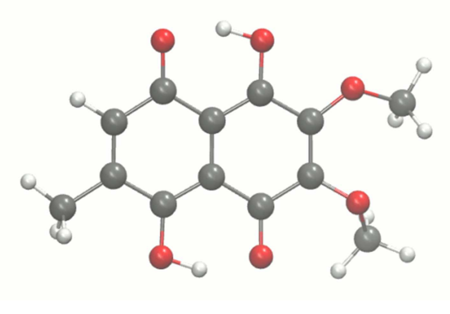	**B3LYP = -598638.9652**<br>***PBE = -597969.6648**<br>**ωB97XD = -598432.6371**
**Proton on the donor side in the Bridge 1 and proton on the acceptor side in the Bridge 2**	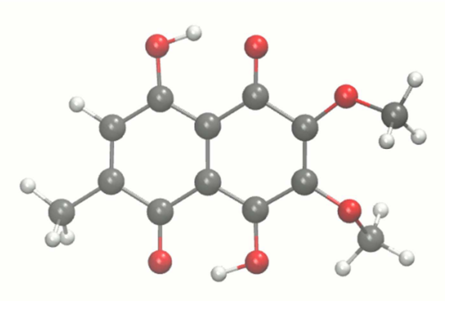	**B3LYP = -598638.9011**<br>***PBE = -597969.6642**<br>**ωB97XD = -598432.5104**
**Proton-transferred form**	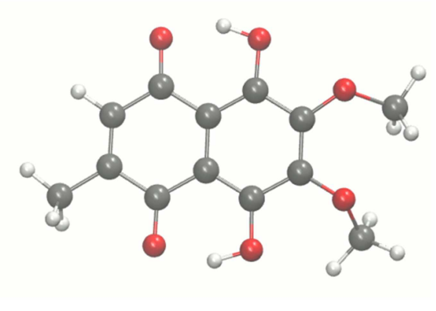	**B3LYP = -598640.9079**<br>**PBE = -597967.3776**<br>**ωB97XD = -598436.2578**

*** Basis set used for the simulations: 6-311++G(2d,2p)**

***O8-H^{BP1}…O1 – Bridge 1**

O5-H^{BP2}…O4 – Bridge 2

*** in case of PBE functional the molecular form was obtained**

Table S2. Selected geometric parameters related to the intramolecular hydrogen bonds of 2,3-dimethylnaphthazarin (**1**) and 2,3-dimethoxy-6-methylnaphthazarin (**2**). Comparison of experimental [1, 2] and computed data. Metric parameters are given in Å and degrees. CPMD results are presented as average ± standard deviation. For atoms numbering scheme see Figure S1.

Metric parameters	Experimental X-ray	Computational methods				
		B3LYP	ωB97XD	PBE	CPMD (gas phase)	CPMD (solid state)
**2,3-dimethylnaphthazarin (1)** [1]						
**O8…O1**	2.605	2.564	2.573	2.525	2.537 ± 0.087	2.555 ± 0.092
**O8-H^{BP1}**	0.740	0.990	0.981	1.022	1.087 ± 0.162	1.084 ± 0.174
**H^{BP1}…O1**	1.984	1.675	1.700	1.576	1.538 ± 0.206	1.580 ± 0.228
**<O8H^{BP1}O1**	141.5	147.2	146.0	152.1	150.28 ± 6.25	146.95 ± 7.10
**O5…O4**	–	2.569	2.577	2.528	2.541 ± 0.090	2.557 ± 0.089
**O5-H^{BP2}**	–	0.990	0.981	1.023	1.086 ± 0.167	1.089 ± 0.181
**H^{BP2}…O4**	–	1.679	1.704	1.577	1.542 ± 0.206	1.575 ± 0.227
**<O5H^{BP2}O4**	–	147.3	146.1	152.2	150.24 ± 6.30	147.40 ± 6.93
**2,3-dimethoxy-6-methylnaphthazarin (2)** [2]						
**O8…O1**	2.589	2.573	2.583	2.534	2.549 ± 0.090	2.484 ± 0.080
**O8-H^{BP1}**	1.025	0.990	0.981	1.021	1.076 ± 0.156	1.322 ± 0.230
**H^{BP1}…O1**	1.796	1.684	1.711	1.587	1.563 ± 0.201	1.229 ± 0.210
**<O8H^{BP1}O1**	131.3	147.2	146.0	152.0	150.01 ± 6.41	153.86 ± 5.65
**O5…O4**	2.551	2.554	2.564	2.518	2.528 ± 0.086	2.470 ± 0.079
**O5-H^{BP2}**	1.018	0.992	0.982	1.024	1.080 ± 0.156	1.241 ± 0.205
**H^{BP2}…O4**	1.590	1.657	1.684	1.564	1.531 ±0.189	1.307 ±0.245
**<O5H^{BP2}O4**	155.3	148.2	147.0	152.8	150.96 ± 6.06	152.00 ± 6.49

***static DFT simulations were performed with 6-311++G(2d,2p) basis set, "–" lack of bridged proton in the molecule.**

***O8-H^{BP1}…O1 – Bridge 1**

O5-H^{BP2}…O4 – Bridge 2

Sets of coordinates for the minima and transition state estimates from the DFT scans (XYZ format).

The geometry was obtained using the Gaussian 16 Rev. A.03 suite of programs [3].

The 6-311++G(2d,2p) basis set was used during the QM simulations.

Compound I (B3LYP functional)

Geometry for the first minimum:

```
26
compound 1
     6  -2.998324   0.673202  -0.000034
     6  -2.993098  -0.696480  -0.000096
     6  -1.775874  -1.413791   0.000033
     6  -0.565964  -0.708616  -0.000052
     6  -0.570265   0.708914  -0.000014
     6  -1.787646   1.402152   0.000142
     6   0.689564   1.441247  -0.000149
     6   1.971813   0.689409  -0.000305
     6   1.970747  -0.666079  -0.000185
     6   0.699107  -1.427692  -0.000272
     8  -1.834112  -2.749503   0.000138
     8   0.733473  -2.671350  -0.000265
     6   3.214578  -1.503480   0.000477
     6   3.231228   1.513788  -0.000190
     8   0.701688   2.685131  -0.000045
     8  -1.861914   2.736932   0.000296
     1  -3.915925  -1.258968  -0.000228
     1  -3.925599   1.228251  -0.000132
     1   3.225874  -2.164255  -0.867393
     1   3.229154  -2.157699   0.873371
     1   4.116539  -0.900754  -0.003189
     1   3.842833   1.297077  -0.876904
     1   3.838839   1.302658   0.880729
     1   2.986387   2.570902  -0.003872
     1  -0.929036   3.068221   0.000299
     1  -0.897334  -3.070567   0.000195
```

Geometry for the transition state:

26
compound 1

6	-2.985253	0.749965	0.000009
6	-3.009032	-0.611880	-0.000014
6	-1.801620	-1.377374	-0.000031
6	-0.573954	-0.721280	0.000005
6	-0.545310	0.693419	0.000028
6	-1.734593	1.463612	0.000025
6	0.678503	1.387231	0.000016
6	1.949629	0.661009	-0.000007
6	1.940784	-0.703224	0.000114
6	0.669582	-1.461578	0.000030
8	-1.898760	-2.705019	-0.000074
8	0.680162	-2.710749	-0.000030
6	3.183359	-1.541086	0.000147
6	3.216972	1.476223	-0.000191
8	0.677104	2.674279	0.000019
8	-1.684362	2.744286	0.000047
1	-3.944326	-1.154509	-0.000035
1	-3.898149	1.328350	0.000008
1	3.195107	-2.197629	-0.871274
1	3.192607	-2.201162	0.868874
1	4.088080	-0.942331	0.002564
1	3.825100	1.255990	-0.878307
1	3.823901	1.258169	0.879328
1	2.987040	2.536685	-0.001579
1	-0.432087	3.053795	0.000023
1	-0.964823	-3.049959	-0.000082

Geometry for the second minimum:

26
compound 1

6	-3.013066	0.722434	0.000004
6	-3.015599	-0.630188	-0.000022
6	-1.785444	-1.375660	-0.000023
6	-0.563582	-0.708116	0.000017
6	-0.542358	0.715138	0.000035
6	-1.767193	1.480741	0.000034
6	0.683155	1.372526	0.000010
6	1.938199	0.647609	-0.000013
6	1.940833	-0.720176	0.000054
6	0.669724	-1.462515	0.000017
8	-1.865483	-2.695797	-0.000065
8	0.653171	-2.719213	-0.000017
6	3.203401	-1.537573	0.000110
6	3.191807	1.476501	-0.000131
8	0.756421	2.692434	-0.000004
8	-1.766419	2.735483	0.000052
1	-3.937708	-1.195302	-0.000045
1	-3.932026	1.291430	0.000005
1	2.957837	-2.594874	0.001006
1	3.814118	-1.324113	0.878685
1	3.813306	-1.325490	-0.879371
1	3.216243	2.131645	-0.872131
1	4.085962	0.862795	-0.000613
1	3.216808	2.131120	0.872246
1	-0.184016	3.058106	0.000003
1	-0.911309	-3.022901	-0.000068

Compound I (ωB97XD functional)

Geometry for the first minimum:

26
compound 1

6	-2.990756	0.669212	-0.000104
6	-2.985034	-0.694758	-0.000046
6	-1.770011	-1.411999	0.000085
6	-0.569670	-0.707905	0.000004
6	-0.574500	0.708096	-0.000013
6	-1.782972	1.399205	-0.000019
6	0.687288	1.440128	-0.000078
6	1.971655	0.686063	-0.000108
6	1.970931	-0.660076	-0.000215
6	0.698047	-1.425235	-0.000132
8	-1.836983	-2.742540	0.000358
8	0.738870	-2.657383	-0.000372
6	3.210901	-1.497549	0.000037
6	3.228574	1.507920	0.000121
8	0.704508	2.672459	0.000169
8	-1.867519	2.728635	-0.000059
1	-3.908145	-1.257113	-0.000038
1	-3.918747	1.223469	-0.000175
1	3.219434	-2.155275	-0.869562
1	3.222802	-2.149538	0.873981
1	4.113216	-0.894333	-0.003479
1	3.835307	1.289804	-0.879201
1	3.833963	1.291229	0.880743
1	2.986258	2.566051	-0.000873
1	-0.948494	3.071104	-0.000053
1	-0.913321	-3.073393	0.000690

Geometry for the transition state:

26
compound 1

6	-2.975593	0.754823	-0.000040
6	-3.001920	-0.599760	-0.000153
6	-1.796926	-1.372929	-0.000120
6	-0.578094	-0.724752	0.000083
6	-0.545552	0.691652	0.000162
6	-1.721916	1.464618	0.000095
6	0.672436	1.383445	0.000115
6	1.947711	0.653880	0.000006
6	1.938599	-0.699811	0.000626
6	0.665204	-1.463874	0.000292
8	-1.912921	-2.694466	-0.000353
8	0.683765	-2.701127	0.000030
6	3.177188	-1.538131	0.000448
6	3.211843	1.467298	-0.000808
8	0.678152	2.658708	0.000138
8	-1.670406	2.738559	0.000158
1	-3.939240	-1.139475	-0.000301
1	-3.887311	1.335499	-0.000080
1	3.191600	-2.182837	-0.879173
1	3.177213	-2.204635	0.863518
1	4.082447	-0.939358	0.014796
1	3.817350	1.242681	-0.879175
1	3.814799	1.248233	0.880781
1	2.982516	2.528352	-0.004279
1	-0.429362	3.055040	0.000248
1	-0.996635	-3.055648	-0.000358

Geometry for the second minimum:

26
compound 1

6	-3.016391	0.690161	0.000060
6	-3.003621	-0.653796	0.000051
6	-1.760695	-1.387238	0.000037
6	-0.557742	-0.710189	-0.000012
6	-0.546153	0.717065	-0.000026
6	-1.776252	1.466329	-0.000025
6	0.662807	1.385729	0.000006
6	1.931870	0.669267	0.000027
6	1.939054	-0.688279	-0.000011
6	0.679232	-1.448365	-0.000027
8	-1.836492	-2.701434	0.000004
8	0.694714	-2.694219	-0.000049
6	3.185374	-1.515448	-0.000027
6	3.199734	1.480891	0.000062
8	0.723545	2.699005	0.000034
8	-1.798387	2.710419	-0.000108
1	-3.918907	-1.230306	0.000090
1	-3.941273	1.249965	0.000120
1	3.197057	-2.171646	-0.871103
1	3.197544	-2.170967	0.871551
1	4.087121	-0.911354	-0.000499
1	3.802503	1.253540	-0.879443
1	3.802122	1.254038	0.879959
1	2.983880	2.543941	-0.000280
1	-0.206536	3.064528	-0.000155
1	-0.893854	-3.028677	0.000025

Compound I (PBE functional)

Geometry for the first minimum:

26
compound 1

6	-3.010568	0.676664	0.000211
6	-3.005215	-0.700831	0.000210
6	-1.779456	-1.421204	-0.000013
6	-0.560547	-0.708563	-0.000192
6	-0.564948	0.708941	-0.000181
6	-1.791584	1.409037	-0.000015
6	0.687559	1.443732	-0.000268
6	1.966570	0.697095	-0.000249
6	1.966087	-0.674357	-0.000279
6	0.697176	-1.430729	-0.000240
8	-1.809409	-2.756757	-0.000057
8	0.718351	-2.695624	0.000165
6	3.212869	-1.505675	0.000138
6	3.225879	1.518859	0.000051
8	0.687002	2.708735	0.000347
8	-1.838239	2.744042	-0.000112
1	-3.935613	-1.268345	0.000341
1	-3.945562	1.236565	0.000338
1	3.224788	-2.175220	-0.872200
1	3.229066	-2.167792	0.878153
1	4.120970	-0.896344	-0.004318
1	3.843592	1.301501	-0.883080
1	3.840703	1.305050	0.886107
1	2.977357	2.584948	-0.002301
1	-0.856312	3.029186	-0.000472
1	-0.823569	-3.030523	-0.000347

Geometry for the transition state:

26
compound 1

6	-3.006984	0.720217	0.000006
6	-3.016139	-0.650903	0.000054
6	-1.792443	-1.398806	-0.000156
6	-0.562808	-0.714490	0.000095
6	-0.550158	0.699400	0.000041
6	-1.762556	1.456301	-0.000370
6	0.678205	1.400659	0.000126
6	1.946837	0.681726	-0.000023
6	1.944067	-0.698560	0.000039
6	0.679311	-1.451310	0.000161
8	-1.837099	-2.727373	-0.000093
8	0.678802	-2.722917	0.000116
6	3.192592	-1.526365	-0.000000
6	3.213065	1.496791	-0.000368
8	0.667541	2.705814	0.000458
8	-1.730077	2.744780	-0.000018
1	-3.952063	-1.210580	0.000116
1	-3.934433	1.292863	0.000057
1	3.206149	-2.194047	-0.874016
1	3.207848	-2.191357	0.876079
1	4.101355	-0.917934	-0.001688
1	3.827182	1.275998	-0.884997
1	3.827473	1.276241	0.884157
1	2.981196	2.566236	-0.000321
1	-0.436605	3.020300	-0.000428
1	-0.839373	-3.008113	-0.000295

Geometry for the second minimum:

26
compound 1

6	-3.020445	0.709012	-0.000029
6	-3.021832	-0.657907	-0.000048
6	-1.789132	-1.397838	-0.000022
6	-0.560843	-0.708424	0.000012
6	-0.551183	0.708713	0.000034
6	-1.777946	1.464710	0.000023
6	0.678517	1.396201	0.000018
6	1.935703	0.676097	-0.000047
6	1.937247	-0.706588	0.000023
6	0.675725	-1.454094	0.000025
8	-1.826233	-2.721869	-0.000056
8	0.658356	-2.730216	0.000016
6	3.213640	-1.492816	0.000101
6	3.217979	1.459924	-0.000167
8	0.695207	2.717092	0.000053
8	-1.767344	2.740997	0.000065
1	-3.952423	-1.226630	-0.000087
1	-3.949952	1.278559	-0.000043
1	2.998232	-2.566108	0.000063
1	3.827049	-1.254058	0.882409
1	3.827284	-1.253938	-0.881981
1	3.827559	1.212843	-0.882267
1	3.827837	1.212752	0.881712
1	3.025455	2.536741	-0.000112
1	-0.322432	3.024651	0.000163
1	-0.813082	-2.994785	-0.000017

Compound II – scan 1 (B3LYP functional)

<u>Geometry for the first minimum</u>:

31
compound 2

6	-0.601404	1.587314	-0.040069
8	-0.713118	2.818773	0.006208
6	-1.833650	0.741433	-0.115846
8	-2.929152	1.499687	-0.152169
6	-4.255422	0.977961	0.013680
1	-4.541304	0.350120	-0.824117
1	-4.336525	0.417106	0.943387
1	-4.888175	1.859072	0.061891
6	-1.739679	-0.621147	-0.159376
8	-2.848524	-1.391592	-0.345876
6	-3.167503	-2.352852	0.676277
1	-2.368306	-3.078178	0.793801
1	-3.362114	-1.843610	1.623238
1	-4.072973	-2.848296	0.337878
6	-0.428098	-1.290066	-0.158322
8	-0.370622	-2.530965	-0.253141
6	2.035240	-1.103869	-0.041652
8	2.177458	-2.434058	-0.106616
1	1.261664	-2.806859	-0.185966
6	3.224612	-0.323686	0.050437
6	4.553291	-1.020306	0.076128
1	5.367690	-0.303186	0.149330
1	4.613386	-1.709559	0.919521
1	4.692641	-1.621536	-0.823366
6	3.113528	1.044661	0.107759
1	3.997410	1.663894	0.175580
6	1.861172	1.693985	0.077152
8	1.847965	3.029987	0.131535
1	0.897304	3.303344	0.097950
6	0.784337	-0.482808	-0.063314
6	0.691450	0.929884	-0.007631

Geometry for the transition state:

31
compound 2

6	-0.572666	1.540845	-0.059435
8	-0.651323	2.818568	-0.019907
6	-1.803204	0.739449	-0.129716
8	-2.902135	1.500397	-0.174918
6	-4.207618	0.997370	0.143540
1	-4.584359	0.348965	-0.640902
1	-4.192982	0.463429	1.092911
1	-4.826865	1.884893	0.233482
6	-1.720971	-0.632450	-0.173399
8	-2.845047	-1.371318	-0.378937
6	-3.174195	-2.380918	0.593812
1	-2.393226	-3.131806	0.655785
1	-3.338123	-1.919862	1.570609
1	-4.099685	-2.829126	0.244126
6	-0.419348	-1.319886	-0.153451
8	-0.353733	-2.566286	-0.237572
6	2.038040	-1.096432	-0.026155
8	2.200401	-2.418523	-0.079975
1	1.284202	-2.803885	-0.158889
6	3.226406	-0.281739	0.065351
6	4.560136	-0.968179	0.103218
1	5.367990	-0.243815	0.173979
1	4.620842	-1.649801	0.952749
1	4.707648	-1.575991	-0.790539
6	3.103025	1.078496	0.109269
1	3.978645	1.709562	0.176052
6	1.821729	1.726412	0.065603
8	1.702861	3.002349	0.101158
1	0.438667	3.251497	0.044605
6	0.780001	-0.508524	-0.058677
6	0.673508	0.901298	-0.018753

Geometry for the second minimum:

31
compound 2

6	-0.581492	1.539796	-0.071011
8	-0.733145	2.848090	-0.039924
6	-1.796514	0.739538	-0.139336
8	-2.907422	1.487779	-0.193535
6	-4.187369	0.989997	0.222309
1	-4.613706	0.324368	-0.521249
1	-4.107274	0.476089	1.179687
1	-4.802834	1.877661	0.335438
6	-1.715743	-0.635160	-0.180954
8	-2.843101	-1.365463	-0.393411
6	-3.165471	-2.403571	0.551560
1	-2.394777	-3.167069	0.569005
1	-3.298354	-1.974860	1.547534
1	-4.106642	-2.823730	0.208882
6	-0.419022	-1.306995	-0.150928
8	-0.338356	-2.561063	-0.229444
6	2.019210	-1.088871	-0.021119
8	2.156744	-2.404366	-0.072576
1	1.217218	-2.770653	-0.151266
6	3.233948	-0.297566	0.071993
6	4.549396	-1.016929	0.115203
1	5.373874	-0.311718	0.186795
1	4.590409	-1.697882	0.966351
1	4.684082	-1.630003	-0.776894
6	3.132688	1.054629	0.110955
1	4.015305	1.676163	0.178358
6	1.857110	1.753363	0.062042
8	1.801641	3.005706	0.093583
1	0.183048	3.256322	0.015271
6	0.767789	-0.484128	-0.056518
6	0.666931	0.935872	-0.023438

Compound II – scan 2 (B3LYP functional)

Geometry for the first minimum:

31
compound 2

6	-0.601404	1.587314	-0.040069
8	-0.713118	2.818773	0.006208
6	-1.833650	0.741433	-0.115846
8	-2.929152	1.499687	-0.152169
6	-4.255422	0.977961	0.013680
1	-4.541304	0.350120	-0.824117
1	-4.336525	0.417106	0.943387
1	-4.888175	1.859072	0.061891
6	-1.739679	-0.621147	-0.159376
8	-2.848524	-1.391592	-0.345876
6	-3.167503	-2.352852	0.676277
1	-2.368306	-3.078178	0.793801
1	-3.362114	-1.843610	1.623238
1	-4.072973	-2.848296	0.337878
6	-0.428098	-1.290066	-0.158322
8	-0.370622	-2.530965	-0.253141
6	2.035240	-1.103869	-0.041652
8	2.177458	-2.434058	-0.106616
1	1.261664	-2.806859	-0.185966
6	3.224612	-0.323686	0.050437
6	4.553291	-1.020306	0.076128
1	5.367690	-0.303186	0.149330
1	4.613386	-1.709559	0.919521
1	4.692641	-1.621536	-0.823366
6	3.113528	1.044661	0.107759
1	3.997410	1.663894	0.175580
6	1.861172	1.693985	0.077152
8	1.847965	3.029987	0.131535
1	0.897304	3.303344	0.097950
6	0.784337	-0.482808	-0.063314
6	0.691450	0.929884	-0.007631

Geometry for the transition state:

31
compound 2

6	-0.601159	1.611112	-0.021433
8	-0.713861	2.846900	0.033687
6	-1.820459	0.747547	-0.107135
8	-2.926322	1.488481	-0.141884
6	-4.253180	0.945685	-0.085013
1	-4.465791	0.330462	-0.953580
1	-4.396952	0.366368	0.825251
1	-4.900092	1.817581	-0.069558
6	-1.710820	-0.622379	-0.154945
8	-2.809063	-1.415299	-0.330986
6	-3.123916	-2.332195	0.731607
1	-2.306742	-3.026813	0.905489
1	-3.354688	-1.783364	1.647708
1	-4.004836	-2.874297	0.400099
6	-0.398452	-1.243808	-0.149008
8	-0.302708	-2.525282	-0.238403
6	2.008526	-1.131026	-0.055138
8	2.057666	-2.412747	-0.132077
1	0.830090	-2.805660	-0.220373
6	3.222850	-0.340079	0.037161
6	4.537940	-1.055322	0.048175
1	5.367783	-0.355911	0.121825
1	4.586567	-1.755313	0.883673
1	4.655833	-1.653903	-0.856384
6	3.117881	1.021236	0.104719
1	4.004641	1.637579	0.171096
6	1.860615	1.693536	0.087755
8	1.860740	3.023866	0.150601
1	0.906874	3.303414	0.123840
6	0.768303	-0.456495	-0.059928
6	0.682821	0.954273	0.005418

Geometry for the second minimum:

31
compound 2

6	-0.617591	1.587042	0.036144
8	-0.726622	2.827993	0.123425
6	-1.827469	0.731684	-0.061877
8	-2.949237	1.452932	-0.064718
6	-4.218554	0.929277	-0.483437
1	-4.127853	0.398630	-1.427495
1	-4.642645	0.269234	0.269057
1	-4.847224	1.806640	-0.603338
6	-1.705669	-0.637736	-0.116279
8	-2.804163	-1.446894	-0.214313
6	-3.102665	-2.237287	0.949444
1	-2.281815	-2.910372	1.186923
1	-3.317749	-1.590513	1.802775
1	-3.986886	-2.814828	0.695424
6	-0.399694	-1.250663	-0.153577
8	-0.357289	-2.565024	-0.262897
6	2.044054	-1.143617	-0.098716
8	2.146784	-2.394507	-0.196735
1	0.619069	-2.835766	-0.271998
6	3.246254	-0.300622	-0.002424
6	4.575164	-0.986371	-0.024971
1	5.391165	-0.271189	0.052228
1	4.648421	-1.703795	0.793710
1	4.690737	-1.564471	-0.942973
6	3.110480	1.047644	0.099264
1	3.978596	1.689980	0.168641
6	1.828795	1.687104	0.116077
8	1.799802	3.007768	0.212316
1	0.827348	3.261451	0.203584
6	0.766127	-0.486959	-0.073994
6	0.663209	0.928312	0.030485

Compound II – scan 1 (ωB97XD functional)

Geometry for the first minimum:

31
compound 2

6	-0.603110	1.581246	-0.049058
8	-0.723532	2.800712	-0.005100
6	-1.836335	0.730869	-0.127550
8	-2.926040	1.484296	-0.156328
6	-4.230443	0.948914	0.038934
1	-4.530149	0.318327	-0.792923
1	-4.279481	0.382605	0.969050
1	-4.878070	1.817917	0.108714
6	-1.739948	-0.621397	-0.179361
8	-2.832181	-1.403453	-0.367546
6	-3.167928	-2.287120	0.699210
1	-2.365703	-2.996296	0.889769
1	-3.390285	-1.717624	1.606105
1	-4.059669	-2.818188	0.378011
6	-0.425062	-1.291018	-0.170655
8	-0.373470	-2.520409	-0.261869
6	2.030013	-1.101757	-0.040266
8	2.183931	-2.425724	-0.101364
1	1.283618	-2.809905	-0.184355
6	3.213992	-0.319231	0.056975
6	4.539134	-1.016358	0.089876
1	5.353961	-0.300242	0.169347
1	4.588947	-1.705587	0.933343
1	4.679452	-1.614657	-0.810892
6	3.103071	1.042807	0.112662
1	3.986913	1.662427	0.184509
6	1.852203	1.691377	0.076080
8	1.846737	3.022117	0.130878
1	0.908810	3.305757	0.094233
6	0.787877	-0.483470	-0.069063
6	0.692885	0.927666	-0.013496

Geometry for the transition state:

31
compound 2

6	-0.568174	1.533021	-0.066441
8	-0.654175	2.798924	-0.026493
6	-1.802754	0.727771	-0.141266
8	-2.894871	1.484541	-0.177063
6	-4.178331	0.967697	0.157961
1	-4.562132	0.318196	-0.623219
1	-4.138304	0.425746	1.103460
1	-4.810993	1.843542	0.268602
6	-1.719354	-0.632846	-0.193630
8	-2.828456	-1.382428	-0.401300
6	-3.177657	-2.308685	0.625678
1	-2.395558	-3.050244	0.767806
1	-3.369210	-1.777920	1.562429
1	-4.091094	-2.792603	0.291315
6	-0.415215	-1.325130	-0.166750
8	-0.358703	-2.559826	-0.246882
6	2.031854	-1.095228	-0.026359
8	2.212290	-2.409943	-0.076712
1	1.314441	-2.810128	-0.158821
6	3.215680	-0.272208	0.071054
6	4.546388	-0.957628	0.115667
1	5.353939	-0.233498	0.192916
1	4.597273	-1.639670	0.964919
1	4.695056	-1.562178	-0.779586
6	3.091324	1.080306	0.114697
1	3.965611	1.713399	0.185870
6	1.807069	1.723818	0.065451
8	1.685666	2.992102	0.101684
1	0.432530	3.248539	0.040611
6	0.783043	-0.515104	-0.065535
6	0.671866	0.895860	-0.024222

Geometry for the second minimum:

31
compound 2

6	-0.574551	1.531656	-0.077081
8	-0.734742	2.829768	-0.044733
6	-1.793314	0.726827	-0.150978
8	-2.897008	1.471274	-0.193881
6	-4.155682	0.959734	0.231277
1	-4.590028	0.300589	-0.514549
1	-4.053479	0.429978	1.179203
1	-4.780948	1.836726	0.372300
6	-1.712558	-0.636723	-0.202033
8	-2.825541	-1.377599	-0.418082
6	-3.171597	-2.326137	0.589505
1	-2.392567	-3.075048	0.706934
1	-3.351354	-1.816933	1.540470
1	-4.091453	-2.795498	0.251865
6	-0.414632	-1.314071	-0.165116
8	-0.342027	-2.556639	-0.239582
6	2.010875	-1.087170	-0.021923
8	2.166375	-2.394842	-0.070477
1	1.246152	-2.775211	-0.152272
6	3.224451	-0.287022	0.077286
6	4.535619	-1.007157	0.126583
1	5.360754	-0.303151	0.203078
1	4.566807	-1.687310	0.978222
1	4.669904	-1.618162	-0.766457
6	3.123397	1.057095	0.116542
1	4.004983	1.680287	0.188146
6	1.842844	1.751873	0.062850
8	1.778646	2.994131	0.095350
1	0.170624	3.251237	0.012311
6	0.768487	-0.491921	-0.063496
6	0.662492	0.930308	-0.028083

Compound II – scan 2 (ωB97XD functional)

<u>Geometry for the first minimum</u>:

31
compound 2

6	-0.603130	1.581240	-0.049060
8	-0.723565	2.800707	-0.005102
6	-1.836347	0.730852	-0.127551
8	-2.926060	1.484268	-0.156329
6	-4.230457	0.948872	0.038934
1	-4.530157	0.318282	-0.792923
1	-4.279489	0.382562	0.969050
1	-4.878093	1.817869	0.108714
6	-1.739943	-0.621412	-0.179361
8	-2.832167	-1.403481	-0.367545
6	-3.167903	-2.287151	0.699211
1	-2.365670	-2.996317	0.889770
1	-3.390267	-1.717657	1.606106
1	-4.059639	-2.818230	0.378013
6	-0.425050	-1.291019	-0.170656
8	-0.373443	-2.520406	-0.261867
6	2.030036	-1.101742	-0.040270
8	2.184060	-2.425703	-0.101365
1	1.283852	-2.810143	-0.184379
6	3.213994	-0.319180	0.056977
6	4.539153	-1.016276	0.089881
1	5.353963	-0.300142	0.169355
1	4.588979	-1.705506	0.933347
1	4.679487	-1.614571	-0.810888
6	3.103051	1.042856	0.112666
1	3.986880	1.662495	0.184515
6	1.852169	1.691391	0.076081
8	1.846619	3.022127	0.130878
1	0.908642	3.305564	0.094225
6	0.787885	-0.483475	-0.069066
6	0.692870	0.927661	-0.013497

Geometry for the transition state:

31
compound 2

6	-0.601366	1.610383	-0.036000
8	-0.724702	2.833884	0.013717
6	-1.820933	0.738958	-0.122822
8	-2.921807	1.473727	-0.153895
6	-4.225696	0.918877	-0.020501
1	-4.482817	0.298369	-0.873994
1	-4.307153	0.338232	0.898109
1	-4.886495	1.778938	0.034455
6	-1.708841	-0.620185	-0.176936
8	-2.790171	-1.422184	-0.360416
6	-3.120138	-2.280459	0.728436
1	-2.303864	-2.964330	0.952464
1	-3.367023	-1.690357	1.615439
1	-3.993804	-2.843779	0.412687
6	-0.390854	-1.241081	-0.159306
8	-0.299708	-2.510902	-0.242923
6	1.996848	-1.129755	-0.049663
8	2.047328	-2.404897	-0.121553
1	0.833305	-2.806699	-0.217402
6	3.210774	-0.339692	0.047658
6	4.519523	-1.060657	0.069282
1	5.352674	-0.365305	0.145206
1	4.555009	-1.755839	0.908807
1	4.635310	-1.661630	-0.833352
6	3.108374	1.014229	0.109859
1	3.996472	1.629063	0.179809
6	1.851395	1.691934	0.083550
8	1.866080	3.016950	0.143032
1	0.927106	3.310634	0.111548
6	0.768246	-0.454040	-0.063970
6	0.683521	0.958165	-0.002499

Geometry for the second minimum:

31
compound 2

6	-0.620604	1.579439	0.026471
8	-0.740577	2.809120	0.112546
6	-1.828546	0.716919	-0.073991
8	-2.945540	1.432199	-0.071934
6	-4.202636	0.888863	-0.461015
1	-4.123200	0.360752	-1.408521
1	-4.598681	0.217818	0.298777
1	-4.855122	1.750841	-0.566008
6	-1.699478	-0.640836	-0.137208
8	-2.777485	-1.465960	-0.234133
6	-3.109739	-2.148799	0.970948
1	-2.283360	-2.773738	1.309184
1	-3.378524	-1.433342	1.752855
1	-3.966408	-2.775252	0.738161
6	-0.387346	-1.253026	-0.162866
8	-0.360933	-2.561621	-0.262832
6	2.044667	-1.144209	-0.095020
8	2.160868	-2.383037	-0.185500
1	0.597227	-2.844824	-0.265660
6	3.243195	-0.289843	0.004211
6	4.570331	-0.973155	-0.010809
1	5.384917	-0.256638	0.069812
1	4.635910	-1.688063	0.810010
1	4.687011	-1.550116	-0.928782
6	3.101117	1.048488	0.101339
1	3.965134	1.696675	0.173575
6	1.811527	1.684406	0.111672
8	1.790122	2.998098	0.206919
1	0.829827	3.259749	0.196639
6	0.767065	-0.493291	-0.079843
6	0.659387	0.926000	0.022681

Compound II – scan 1 (PBE functional)

Geometry for the first minimum:

31
compound 2

6	-0.597051	1.596566	-0.057268
8	-0.694349	2.850597	-0.020207
6	-1.827453	0.758278	-0.123284
8	-2.931785	1.518949	-0.156279
6	-4.238300	0.979505	0.122570
1	-4.588145	0.335661	-0.690581
1	-4.234391	0.419762	1.067687
1	-4.880889	1.860817	0.218526
6	-1.739026	-0.622285	-0.167303
8	-2.860704	-1.375408	-0.380952
6	-3.169341	-2.388973	0.602949
1	-2.343538	-3.099563	0.711603
1	-3.400829	-1.915006	1.570443
1	-4.060030	-2.900647	0.223743
6	-0.430439	-1.290847	-0.150369
8	-0.358084	-2.552394	-0.242932
6	2.034647	-1.111431	-0.026738
8	2.152638	-2.443679	-0.085255
1	1.187538	-2.776142	-0.166935
6	3.233888	-0.329353	0.062353
6	4.557448	-1.033646	0.095920
1	5.384497	-0.318314	0.167030
1	4.610250	-1.726061	0.948561
1	4.694847	-1.649009	-0.805004
6	3.125618	1.047877	0.106996
1	4.019380	1.669632	0.172596
6	1.866996	1.702069	0.065411
8	1.829721	3.037853	0.105004
1	0.834969	3.264596	0.063733
6	0.775510	-0.481958	-0.055167
6	0.686977	0.932018	-0.015479

<u>Geometry for the transition state</u>:

```
31
compound 2
     6  -0.579598   1.556632  -0.070230
     8  -0.656827   2.852635  -0.037091
     6  -1.804844   0.757063  -0.132146
     8  -2.914356   1.517759  -0.171810
     6  -4.200946   0.991685   0.207725
     1  -4.609573   0.334469  -0.566467
     1  -4.133130   0.449760   1.161067
     1  -4.832229   1.877767   0.331609
     6  -1.723701  -0.632303  -0.174843
     8  -2.858272  -1.359237  -0.397909
     6  -3.167797  -2.413363   0.543486
     1  -2.354279  -3.143511   0.599972
     1  -3.368035  -1.980937   1.536836
     1  -4.077810  -2.885957   0.159676
     6  -0.423649  -1.310772  -0.144458
     8  -0.338364  -2.578408  -0.228173
     6   2.035257  -1.104210  -0.017361
     8   2.160222  -2.429425  -0.069736
     1   1.183977  -2.766002  -0.150091
     6   3.236014  -0.300153   0.071201
     6   4.561444  -1.000829   0.112351
     1   5.385550  -0.282187   0.181849
     1   4.613360  -1.688445   0.968878
     1   4.702825  -1.620608  -0.784918
     6   3.120353   1.070398   0.107426
     1   4.009220   1.699539   0.172505
     6   1.841464   1.733956   0.057774
     8   1.734702   3.019202   0.085594
     1   0.431380   3.226496   0.024850
     6   0.770517  -0.496091  -0.051551
     6   0.674136   0.914555  -0.023168
```

Geometry for the second minimum:

31
compound 2

6	-0.585233	1.553709	-0.075374
8	-0.700485	2.867264	-0.045739
6	-1.801904	0.756709	-0.136665
8	-2.917646	1.511188	-0.181041
6	-4.191368	0.987009	0.242287
1	-4.621893	0.321628	-0.512890
1	-4.093959	0.454454	1.198405
1	-4.820718	1.873019	0.376657
6	-1.720728	-0.634396	-0.177945
8	-2.856837	-1.357254	-0.402758
6	-3.161036	-2.425726	0.524363
1	-2.352849	-3.163208	0.555294
1	-3.341603	-2.009892	1.528402
1	-4.081365	-2.882667	0.146257
6	-0.422678	-1.303165	-0.142727
8	-0.328374	-2.575591	-0.223663
6	2.026309	-1.100511	-0.015722
8	2.137277	-2.421294	-0.067804
1	1.141730	-2.747502	-0.148100
6	3.240057	-0.308062	0.073973
6	4.556181	-1.025294	0.117248
1	5.388681	-0.316568	0.188003
1	4.597757	-1.713540	0.973777
1	4.690873	-1.646822	-0.779798
6	3.134874	1.058191	0.108382
1	4.026984	1.682993	0.173944
6	1.859132	1.749852	0.056617
8	1.786767	3.021896	0.083139
1	0.281480	3.228364	0.010795
6	0.764795	-0.482569	-0.050580
6	0.671476	0.932598	-0.025160

Compound II – scan 2 (PBE functional)

Geometry for the first minimum:

31
compound 2

6	-0.597051	1.596566	-0.057268
8	-0.694349	2.850597	-0.020207
6	-1.827453	0.758278	-0.123284
8	-2.931785	1.518949	-0.156279
6	-4.238300	0.979505	0.122570
1	-4.588145	0.335661	-0.690581
1	-4.234391	0.419762	1.067687
1	-4.880889	1.860817	0.218526
6	-1.739026	-0.622285	-0.167303
8	-2.860704	-1.375408	-0.380952
6	-3.169341	-2.388973	0.602949
1	-2.343538	-3.099563	0.711603
1	-3.400829	-1.915006	1.570443
1	-4.060030	-2.900647	0.223743
6	-0.430439	-1.290847	-0.150369
8	-0.358084	-2.552394	-0.242932
6	2.034647	-1.111431	-0.026738
8	2.152638	-2.443679	-0.085255
1	1.187538	-2.776142	-0.166935
6	3.233888	-0.329353	0.062353
6	4.557448	-1.033646	0.095920
1	5.384497	-0.318314	0.167030
1	4.610250	-1.726061	0.948561
1	4.694847	-1.649009	-0.805004
6	3.125618	1.047877	0.106996
1	4.019380	1.669632	0.172596
6	1.866996	1.702069	0.065411
8	1.829721	3.037853	0.105004
1	0.834969	3.264596	0.063733
6	0.775510	-0.481958	-0.055167
6	0.686977	0.932018	-0.015479

Geometry for the transition state:

31
compound 2

6	-0.596088	1.610750	-0.047943
8	-0.688330	2.870861	-0.006335
6	-1.816189	0.763890	-0.122883
8	-2.928872	1.510919	-0.159606
6	-4.237157	0.955817	0.074869
1	-4.553995	0.312562	-0.752214
1	-4.258060	0.391953	1.017317
1	-4.891965	1.829686	0.154282
6	-1.716401	-0.623578	-0.170015
8	-2.829817	-1.394736	-0.382395
6	-3.146394	-2.365408	0.640001
1	-2.314699	-3.059963	0.802089
1	-3.404879	-1.853406	1.580902
1	-4.020029	-2.909833	0.266784
6	-0.410526	-1.253049	-0.143927
8	-0.314425	-2.552680	-0.233177
6	2.021409	-1.139182	-0.033621
8	2.079816	-2.428365	-0.101292
1	0.807767	-2.783131	-0.196516
6	3.236482	-0.337315	0.056146
6	4.551993	-1.050391	0.080657
1	5.388726	-0.346371	0.153243
1	4.595650	-1.751816	0.926597
1	4.674911	-1.664455	-0.823453
6	3.129167	1.034084	0.107073
1	4.022553	1.657561	0.171528
6	1.864754	1.700122	0.073948
8	1.828115	3.030028	0.117184
1	0.821577	3.253724	0.078052
6	0.765924	-0.467277	-0.050697
6	0.679781	0.944082	-0.005884

Geometry for the second minimum:

31
compound 2

6	-0.596980	1.602310	-0.043332
8	-0.684883	2.866207	0.002505
6	-1.816032	0.762331	-0.121269
8	-2.928181	1.510173	-0.157548
6	-4.242528	0.952478	0.030465
1	-4.530767	0.312569	-0.809618
1	-4.295564	0.384915	0.969353
1	-4.900501	1.825449	0.091158
6	-1.713430	-0.626127	-0.168649
8	-2.825636	-1.404183	-0.370770
6	-3.152047	-2.338607	0.681141
1	-2.322615	-3.027993	0.875879
1	-3.418956	-1.795717	1.602164
1	-4.021740	-2.896721	0.319018
6	-0.411430	-1.251636	-0.148481
8	-0.340342	-2.567706	-0.245281
6	2.037456	-1.149209	-0.040452
8	2.118840	-2.424345	-0.110067
1	0.696628	-2.797222	-0.215034
6	3.248004	-0.324127	0.053821
6	4.567994	-1.027169	0.076696
1	5.400062	-0.317779	0.150822
1	4.615133	-1.730960	0.920499
1	4.693612	-1.639513	-0.828181
6	3.129758	1.042613	0.109695
1	4.015828	1.676443	0.176589
6	1.856260	1.696935	0.078855
8	1.807579	3.022532	0.126426
1	0.788808	3.235066	0.086883
6	0.767429	-0.480861	-0.055022
6	0.675721	0.932740	-0.003744

References:

[1] Rodríguez, J.G.; Smith-Verdier, P.; Florencio, F.; García-Blanco, S. Crystal and molecular structure of 2,3-dimethylnaphthazarin: a charge-transfer complex. *J. Mol. Struct*. **1984**, *112*, 101-109.

[2] Cannon, J.R.; Matsuki, Y.; Patrick, V.A.; White, A.H. Selective O-Demethylation of 5,8-Dihydroxy-2,3-dimethoxy-6-methylnaphtho-1,4-quinone. The Crystal Structures of 5,8-Dihydroxy-2,3-dimethoxy-6-methylnaphtho-1,4-quinone and 2,5,8-Triacetoxy-3-methoxy-6-methylnaphtho-1,4-quinone. *Aust. J. Chem.* **1987**, *40*, 1191-200.

[3] Frisch, M.J.; Trucks, G.W.; Schlegel, H.B.; Scuseria, G.E.; Robb, M.A.; Cheeseman, J.R.; Scalmani, G.; Barone, V.; Petersson, G.A.; Nakatsuji, H.; Li, X.; Caricato, M.; Marenich, A.V.; Bloino, J.; Janesko, B.G.; Gomperts, R.; Mennucci, B.; Hratchian, H.P.; Ortiz, J.V.; Izmaylov, A.F.; Sonnenberg, J.L.; Williams-Young, D.; Ding, F.; Lipparini, F.; Egidi, F.; Goings, J.; Peng, B.; Petrone, A.; Henderson, T.; Ranasinghe, D.; Zakrzewski, V.G.; Gao, J.; Rega, N.; Zheng, G.; Liang, W.; Hada, M.; Ehara, M.; Toyota, K.; Fukuda, R.; Hasegawa, J.; Ishida, M.; Nakajima, T.; Honda, Y.; Kitao, O.; Nakai, H.; Vreven, T.; Throssell, K.; Montgomery, Jr., J.A.; Peralta, J.E.; Ogliaro, F.; Bearpark, M.J.; Heyd, J.J.; Brothers, E.N.; Kudin, K.N.; Staroverov, V.N.; Keith, T.A.; Kobayashi, R.; Normand, J.; Raghavachari, K.; Rendell, A.P.; Burant, J.C.; Iyengar, S.S.; Tomasi, J.; Cossi, M.; Millam, J.M.; Klene, M.; Adamo, C.; Cammi, R.; Ochterski, J.W.; Martin, R.L.; Morokuma, K.; Farkas, O.; Foresman, J.B.; Fox, D.J. Gaussian~16 Revision A.03, 2016. Gaussian Inc. Wallingford CT.
